# The proteomic landscape of genome-wide genetic perturbations

**DOI:** 10.1016/j.cell.2023.03.026

**Published:** 2023-04-19

**Authors:** Christoph B. Messner, Vadim Demichev, Julia Muenzner, Simran K. Aulakh, Natalie Barthel, Annika Röhl, Lucía Herrera-Domínguez, Anna-Sophia Egger, Stephan Kamrad, Jing Hou, Guihong Tan, Oliver Lemke, Enrica Calvani, Lukasz Szyrwiel, Michael Mülleder, Kathryn S. Lilley, Charles Boone, Georg Kustatscher, Markus Ralser

**Affiliations:** 1 The Francis Crick Institute, Molecular Biology of Metabolism Laboratory, London NW1 1AT, UK; 2 Precision Proteomics Center, Swiss Institute of Allergy and Asthma Research (SIAF), University of Zurich, 7265 Davos, Switzerland; 3 Charité Universitätsmedizin Berlin, Department of Biochemistry, 10117 Berlin, Germany; 4 Department of Biochemistry, Cambridge Centre for Proteomics, University of Cambridge, Cambridge CB2 1QW, UK; 5 Charité Universitätsmedizin, Core Facility - High Throughput Mass Spectrometry, 10117 Berlin, Germany; 6 Department of Molecular Genetics, University of Toronto, Toronto, ON M5S3E1, Canada; 7 The Donnelly Centre, University of Toronto, Toronto, ON M5S3E1, Canada; 8 RIKEN Center for Sustainable Resource Science, Wako, 351-0198 Saitama, Japan; 9 Wellcome Centre for Cell Biology, University of Edinburgh, Max Born Crescent, Edinburgh EH9 3BF, Scotland, UK; 10 The Wellcome Centre for Human Genetics, Nuffield Department of Medicine, University of Oxford, Oxford OX3 7BN, UK; 11 Max Planck Institute for Molecular Genetics, 14195 Berlin, Germany

## Abstract

Functional genomic strategies have become fundamental for annotating gene function and regulatory networks. Here, we combined functional genomics with proteomics by quantifying protein abundances in a genome-scale knockout library in *Saccharomyces cerevisiae*, using data-independent acquisition mass spectrometry. We find that global protein expression is driven by a complex interplay of (1) general biological properties, including translation rate, protein turnover, the formation of protein complexes, growth rate, and genome architecture, followed by (2) functional properties, such as the connectivity of a protein in genetic, metabolic, and physical interaction networks. Moreover, we show that functional proteomics complements current gene annotation strategies through the assessment of proteome profile similarity, protein covariation, and reverse proteome profiling. Thus, our study reveals principles that govern protein expression and provides a genome-spanning resource for functional annotation.

## Graphical abstract

**Graphical abstract:**
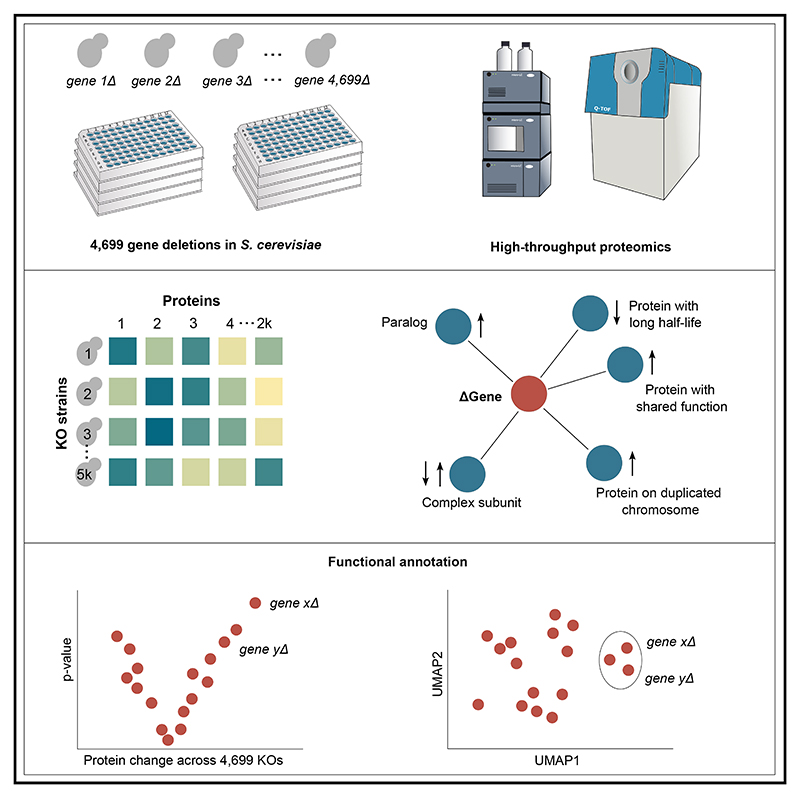

## Introduction

Understanding how genotype leads to phenotype is crucial for molecular biology, biotechnology, synthetic biology, and precision medicine. Predicting the phenotype of a mutant requires knowledge of protein network responses and functions.^1–3^ However, many proteins still lack functional annotation.^4^

Functional genomics, aided by genome editing, has become an essential tool for studying protein function and genetic perturbations. The *S. cerevisiae* knockout (KO) strain collection pioneered functional genomic experiments,^5,6^ enabling the study of genetic and chemical interactions, drug resistance, and their impact on genome and phenome.^7–13^ Integrating systematic gene deletion, transcriptomics, and metabolomics has enabled the characterization of unknown genes using guilt-by-association approaches, providing functional information based on molecular relationships between the gene deletion mutants.^14,15^

The impact of systematic genetic perturbations on the proteome remains less well understood. Until recently, it was challenging to apply proteome technologies at a genome-wide scale. However, proteomes were measured for specific strain collections, such as those focused on mitochondrial function,^16^ deubiquitinating enzymes,^17^ kinases,^18,19^ or metabolic enzymes.^20^ Recent proteomic developments, including robust chromatographic regimes, streamlined sample preparation strategies, and data-independent acquisition,^21–31^ allow for determining the proteome of thousands of samples with high precision and minimal missing values. Such methods have been recently applied for the consistent quantification of almost 1,000 proteins in more than 3,000 gene KOs in *Schizosaccharomyces pombe*^32^ and characterization of the yeast isolates of the 1,011 genomes project.^27^

To understand the proteomic landscape of genome-wide genetic perturbations, we measured quantitative proteomes for a genome-spanning collection of non-essential gene deletions in *Saccharomyces cerevisiae*. We thus created a large, systematic, and quantitative proteomic dataset, with an average of 2,520 proteins quantified across 4,699 yeast gene KO strains. The proteome profiles (PPs) comprise over 100 million peptide quantitations and 9 million protein quantitations. These link deleted genes to proteins and provide a genome-scale resource of molecular phenotypes for 79% of the coding yeast genome. We derive general principles that govern protein expression from the data and demonstrate how functional proteomics reveals gene function.

## Results

### Quantitative proteomes for gene KOs at a genome-wide scale

**Figure 1:**
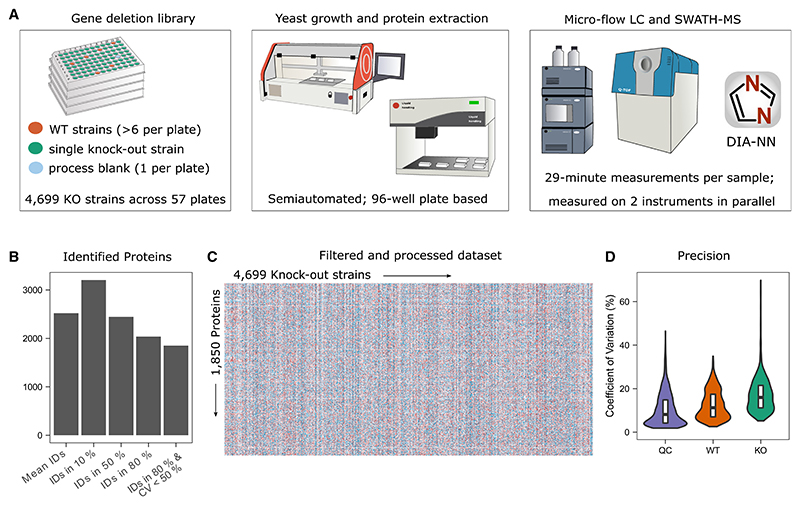
Quantitative proteomes for the genome-scale yeast gene-deletion collection (A) Experimental setup (STAR Methods). (B) Protein identification numbers as mean per sample (2,520), identified in 10% of the samples (3,205), identified in 50% of the samples (2,445), identified in 80% of the samples (2,036), and identified in 80% of the WT samples with CV <50% (filtered dataset as described in STAR Methods) (1,850). All values were calculated for samples that passed the quality control (QC) thresholds. (C) The filtered quantitative data are shown as a heatmap with 1,850 unique proteins measured across the 4,699 KOs, containing 8,693,150 protein quantities. (D) The coefficients of variation (CVs; in %) were calculated for each protein and are shown for pooled yeast digest samples (QC, n = 389), whole-process control samples (WT, n = 388), and KO samples (KO, n = 4,699). Median CV values are 8.1% across the technical replicates of a pooled digest, 11.3% across the biological replicates of the wild-type strain, and 16.2% across the KO library. CVs were calculated on the filtered dataset and are shown from 0% to 70% (see Figure S1B for all data points).

We grew a prototrophic derivative of the yeast gene deletion collection in a synthetic minimal (SM) medium without amino acid and nucleobase supplementation, extracted proteins, and measured the proteomes with an adapted microflow-SWATH-MS approach (Figure 1A; STAR Methods).

The average number of quantified precursors per sample was 20,859, resulting in the average quantification of 2,520 proteins per sample. In total, 3,205 proteins were measured in at least 10% of the samples (Figure 1B). We applied stringent filtering and obtained a map of consistently quantified proteins. This map contains more than 100 million peptide quantities mapped to 8,693,150 protein quantities, providing information on 1,850 unique proteins across the 4,699 measured KOs (Figure 1C; STAR Methods).

In this filtered dataset, the median protein coefficient of variation (CV) was 8.1% for pooled digests (n = 389; reflecting technical variation) and 11.3% for the WT replicates (n = 388; reflecting both technical and biological variation). This variation of our workflow was lower than the biological responses in the KOs, indicated by higher average CV values (16.2% for KOs) (Figures 1D and S1B).

We conducted several analyses to ensure the quality of our dataset. First, we compared the average of the intensities with absolute protein copy numbers obtained by stable-isotope-labeling^33^ and obtained a strong correlation (r = 0.75; Figure S1C). Next, we used the proteomes to validate the yeast KO collection.^5,15^ In 91% of the 960 strains in which the deleted gene was also among the proteins quantified, the *bona fide* deleted gene product was not detected (87%) or was at significantly reduced levels (4%). Of the remaining strains, 37 (4%) had a PP similar to WT strains. In 44 strains, we detected the supposedly deleted gene at wild-type levels, although the proteome differed from the wild-type strain, suggesting that unknown mutations may cause these observed phenotypes (Figures S1D–S1F).

### Protein abundance changes across genome-wide genetic perturbations

Next, we addressed the relationship between protein function and protein abundance changes. We applied linear modeling and empirical Bayes to identify proteins that were differentially expressed (STAR Methods). Based on the repeated measurements of the wild-type proteome, we estimated that our analysis detects 55% of the proteins that are changed 1.5-fold and 84% of the proteins that are changed 2-fold (Figures S1G–S1I).

**Figure 2:**
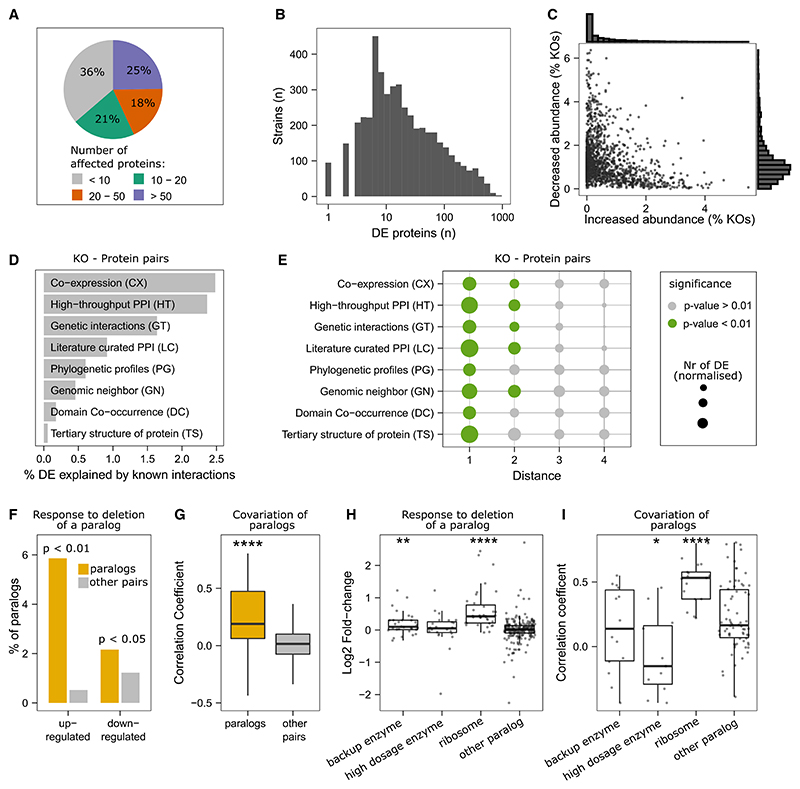
The proteomic response to systematic gene deletion (A) Fraction of gene deletion strains (n = 4,699) in which proteins are differentially expressed (STAR Methods). (B) Distribution of proteomic responses, given as the number of differentially expressed proteins (DE; Benjamini-Hochberg (BH)-adjusted p value < 0.01). (C) Increased and decreased abundance of each protein across the 4,699 KO strains are given as dots and as histograms. (D) Differentially expressed proteins upon gene deletions were compared with physical, genetic, or functional interactions, collected as part of the YeastNet resource (v3).^34^ (E) Differential abundance of proteins is related to their distance to the deleted gene in the indicated network. Differentially abundant proteins of distance *i* were normalized to the total number of proteins of distance *i* within the respective network. A significant enrichment (hypergeometric test, p value < 0.01) is indicated by color. (F) Percentage of paralogs from whole-genome duplications (ohnologs)^35^ that have increased or decreased abundance (BH-adjusted p value < 0.01) after the deletion of one of the paralog partners (yellow). The number of increased or decreased proteins across all KOs is shown as a gray bar for reference. (G) Spearman correlation coefficients are shown for ohnologs^35^ (n = 107 pairs) and for all other protein pairs (n = 1,710,215). The median correlation coefficients are 0.19 and 0.01 for paralogs and other pairs, respectively (Wilcoxon signed-rank test; ****p value ≤ 0.0001). (H) paralogs were classified as compensatory enzymes (backup); enzymes duplicated to increase gene dosage^36^; or protein components of the ribosome (according to the GO term “structural constituent of ribosome^37^”), and compared with measured paralogs not categorized according to these groups (“other paralogs”) (**p value ≤ 0.01; ****p value ≤ 0.0001, Student’s t test). (I) Correlation coefficients are based on Spearman rank coefficients and compared to measured paralogs not categorized (“other paralogs”) (*p value ≤ 0.05; ****p value ≤ 0.0001; Student’s t test). See also Figure S2.

More than 10 proteins were differentially expressed in 64% of the strains, more than 20 in 43%, and more than 50 in 25% (Figures 2A and 2B). The strongest response was detected in *sch9Δ* with 872 of the 1,850 quantified proteins being differentially abundant.

Next, we estimated the impact of the genetic background. We recreated a subset of the KOs in auxotrophic strains used in the synthetic genetic array (SGA) analysis^38^ (STAR Methods). For many KO strains, we found similar protein responses; however, some of the proteome profiles diverged. For instance, Spearman correlation coefficients ranged from ρ = 0.72 for the *dep1Δ* deletion strains to ρ = −0.19 for the *paf1Δ* deletion strain proteomes (Figures S1J–S1L).

### Differential protein expression associated with protein properties and function

Our dataset reveals details about the general nature of differential protein expression. For instance, we report that an individual protein is more often decreased (on average in 1.2% of all KOs) than increased (on average in 0.5% of all KOs). Moreover, individual proteins change predominantly in one direction (Figure 2C). For example, Tsl1 or Tps2, both subunits of the trehalose-6-P synthase, are downregulated in >300 KOs while being increased in only a few strains (Figure S2A). On the other hand, the tRNA synthetases Krs1, Hts1, and Frs1 are primarily increased (Figure S2A).

Next, we aimed to define principal pathways and mechanisms that explained differential protein abundance. We started with a comparison of our data with physical and functional interactions among genes, as annotated in the YeastNet database.^34^ We found that about 8.7% of differential protein expression affects proteins that are directly connected to the deleted gene in these networks (Figure 2D), which represents a significant enrichment (Figure 2E). For example, 2.5% of the differentially expressed proteins are connected with the knocked-out gene in a transcriptional co-expression network or 2.4% in a high-throughput protein-protein interaction network (Figure 2D). In some instances, secondary interactions were also significantly enriched, but 3rd-order interactions were not (Figure 2E). Physical and functional interactions are thus important to explain differential protein expression. Equally, this result also shows that the major fraction of differential protein expression is not explained by the neighborhood of a gene in the functional networks as they are mapped to date.

Another cause of protein abundance changes is functional complementation. We thus investigated the interdependency of paralogs that arose by whole-genome duplication (ohnologs).^35^ In 2.2% and 5.9% of the cases where a paralog was deleted, the other paralog was decreased or increased in abundance, respectively, which is significantly more than the average non-paralog gene pair (p < 0.05; hypergeometric test) (Figure 2F; Table S3). Furthermore, many paralogs have a high level of protein correlation, with 21% having a correlation coefficient (Spearman) larger than 0.5 (Figure 2G). Ribosomal paralogs were particularly interdependent (Figure 2H) and covaried (Figure 2I).

The analysis of metabolic enzymes allowed us to substantiate this picture. We compared our data with a classification of paralog enzymes derived from a genome-scale metabolic network analysis.^36^ We found that paralog enzymes that were classified as having a backup function were significantly increased in abundance on the deletion of the paralog (Figure 2H). On the other hand, paralogs that were classified as high dosage (duplicated enzymes could increase activity and fluxes^36^) have significantly lower correlation coefficients compared to measured paralogs that were not categorized (p = 0.041) (Figure 2I).

### Mapping a complex relationship of growth rate, proteomic changes, and genome versatility

Hence, only a moderate proportion of the overall differential protein abundances was explained by the known functional associations or protein orthology. This could simply mean that the current functional networks (Figure 2D) are incompletely described; this result could however also indicate that most abundance changes are driven by other factors. For example, although the KO strain for *ARG81*, a transcription factor that represses arginine anabolism,^39^ specifically affects proteins involved in arginine metabolism (i.e., Arg8, Arg3, Arg5, Arg56, and Arg1; Figure S2C), other PPs indicate more general perturbations. For instance, the KO of *RPS27B*, encoding for a protein of the small ribosomal subunit (40S), affects the abundance of 91 proteins. A subset of these are functionally related to Rps27b, but in addition, other proteins appear differentially expressed due to Rps27b’s role in the translation itself (Figure S2C). Indeed, KOs of genes that directly or indirectly perturb translation or transcription by having Gene Ontology (GO) annotations such as “ribosomal small subunit progenesis,” “transcription from RNA polymerase I promotor,” or “DNA-templated transcription, termination” generally induce broad proteome changes with a high number of differentially expressed proteins (Figure S2D).

**Figure 3:**
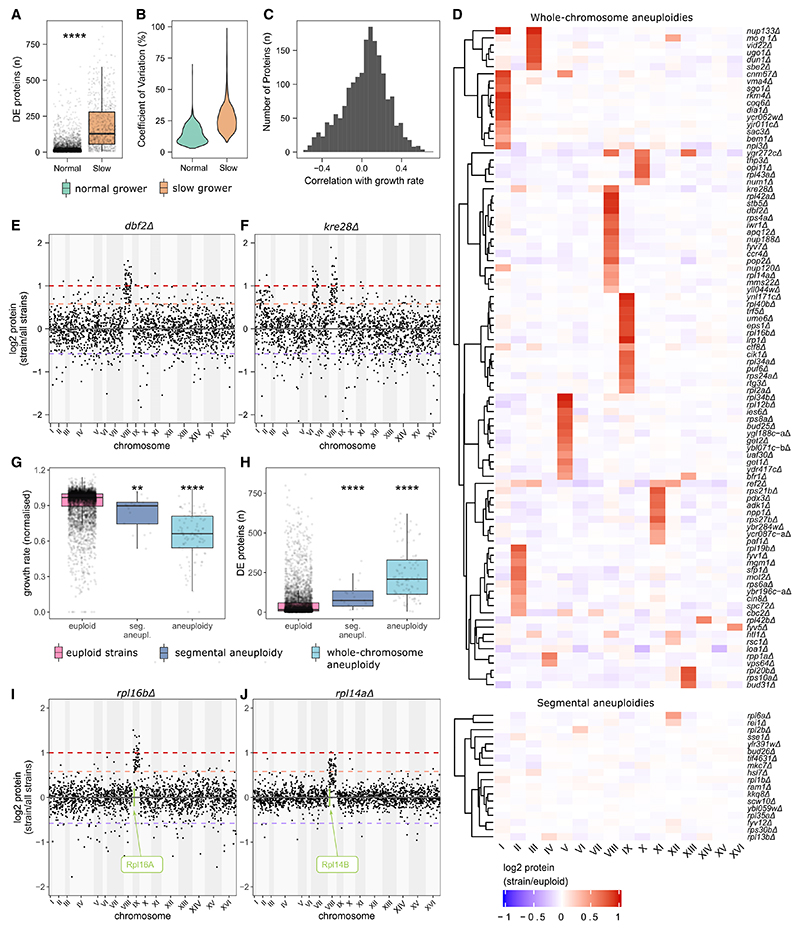
The effect of growth and chromosomal copy-number variations (aneuploidies) on the proteome (A) Numbers of differentially expressed proteins in slow-growing KO strains (n = 748) and normal growers (n = 3,930). ****p value ≤ 0.0001 (Wilcoxon signed-rank test). (B) The proteome dispersion within slow-growing strains is compared with the dispersion within normal-growing strains and is given as protein coefficients of variations (in %). The CV values are shown for CV < 100%. (C) Correlation coefficients (Pearson correlation) are shown as histograms for all pairwise protein-abundance-growth correlations. (D) Median log_2_ protein abundance levels (normalized, see STAR Methods) are shown for each chromosome. (E and F) Protein abundances, sorted by their chromosomal location, are shown for *dbf2Δ* and *kre28Δ*, respectively (Manhattan plot). (G) The normalized growth rates are compared between euploid (n = 4,428, median = 0.97), segmental aneuploidy (n = 18, median = 0.90), and whole-chromosomal aneuploidy strains (n = 84, median = 0.65) (Wilcoxon signed-rank test; **p value ≤ 0.01; ****p value ≤ 0.0001). (F) The numbers of significantly changed proteins are compared between euploid (n = 4,428, median = 16), segmental aneuploidy (n = 18, median = 74), and whole-chromosomal aneuploidy strains (n = 84, median = 208) (Wilcoxon signed-rank test; ****p value ≤ 0.0001). (I and J) Protein abundances, sorted by their chromosomal location, are shown for *rpl16bΔ* and *rpl14aΔ*, respectively. See also Figure S3.

Furthermore, the growth rate is known to affect gene expression. In agreement with previous studies,^14,40–46^ we find that slow-growing strains have a high number of differentially expressed proteins (Figures 3A and 3B). Indeed, the proteome was predictive of growth rates using a random forest (RF) model (R^2^ = 0.68, Figure S3A; STAR Methods). Furthermore, the group of slow-growing strains with broad PPs is dominated by KOs of ribosomal subunits, indicating that the impact on transcription and translation overlaps with the impact of growth rate on the proteome (Figures S2D and S3B).

Conversely, our data also revealed that growth-rate-associated proteins explain only a fraction of differential protein expression in slow-growing strains (Figures 3C and S3C). We realized that one source of divergent profiles is aberrant chromosome numbers (aneuploidies). Aneuploidies cause broad expression changes since all proteins encoded on an aneuploid chromosome are affected.^47–49^ At least in the strain background used herein, aneuploidies are transmitted to transcriptome and proteome with a minimum amount of gene-dosage buffering, rendering aneuploidies discoverable by proteomics.^27,47,50,51^ Sorting protein expression values according to chromosomal localization identified 92 strains with a PP that corresponded to a chromosomal aneuploidy (Figure 3D). For instance, the proteome of the deletion strain for the cell-cycle protein kinase gene *DBF2* reveals duplicated gene doses for proteins encoded on chromosome VIII (Figure 3E). Segmental aneuploidies or short structural aneuploidies were detected for a further 18 strains, often in conjunction with whole-chromosome aneuploidies (Figure 3D). For instance, the deletion strain of the spindle pole body component *KRE28* carries whole-chromosome aneuploidies on chromosomes II and VIII, as well as a segmental aneuploidy on chromosome VII (Figure 3F). We observed all chromosomes except for VI and VII to be aneuploid at least once. Chromosomes IX, VIII, V, and I were aneuploid most frequently (Figure S3D). Aneuploidies on chromosomes VI and VII might be detrimental, and indeed, Chr VI aneuploidy was previously reported to be lethal due to α-tubulin (*TUB2*) being encoded on that chromosome.^52^

Our dataset indicates that aneuploidy is a cause of broad proteomic responses in slow-growing strains. As in laboratory-engineered aneuploids,^47,50^ the aneuploids detected by our approach had slow growth rates (Figure 3G). Furthermore, these strains had broad PPs (Figure 3H). This result was robust on excluding the proteins in the duplicated chromosomes (Figures S3E and S3F).

We next asked whether there is a functional relationship between the deleted gene and the proteomic response in aneuploid strains. Overall, aneuploid strains were enriched for gene deletions in ribosomal proteins as well as proteins involved in the cell cycle and transcription (Figure S3G). In agreement with transcriptomics^53^ and whole-genome resequencing,^54^ we found that KOs of ribosomal subunits, often encoded by two near-identical paralogs,^54^ show compensatory chromosomal duplications. In our dataset, these explain 17 out of 18 aneuploidies found for aneuploid ribosomal gene KOs. In many cases, the aneuploidy results in an increased abundance of the paralog (Figure S3H). For example, *rpl16bΔ* or *rpl14aΔ* cause aneuploidies of chromosomes IX and VIII, respectively, where their paralogs, Rpl16a and Rpl14b, respectively, reside (Figures 3I and 3J). The expression levels of Rpl16a and Rpl14b are increased by fold-changes of 2.15 (adjusted p value = 5.7 × 10^−46^) and 1.77 (adjusted p value = 2.6 × 10^−6^), respectively. Interestingly, the reciprocal Kos (*rpl16aΔ* and *rpl14bΔ*) do not obtain aneuploidies. These situations might indicate divergence in a major and a minor paralog. Indeed, the median intensities are higher in the aneuploidy-inducing paralogs (936 normalized counts per peak [cpp]/2,325 cpp for Rpl16a/Rpl16b and 1,658 cpp/1,063 cpp for Rpl14a/Rpl14b). A second contributing factor is that the frequency of aneuploidies is not equal for all chromosomes.^47^ For instance, Rpl14b and Rpl16a are encoded on chromosomes VIII and IX, which are often aneuploid (in our dataset, in 17 and 14 strains, respectively). Their paralogs instead are located on chromosomes XI and XIV, which are only duplicated in 9 strains and 1 strain, respectively (Figure S3D).

### The effect of protein turnover and ribosome occupancy on differential protein expression

**Figure 4:**
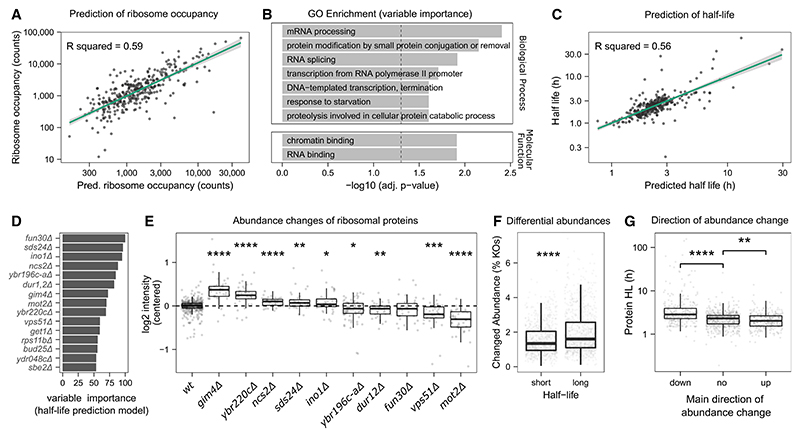
The interdependency of differential protein expression with translation rate and turnover (A) Ribosomal occupancies are predicted with an elastic net model. The model was trained on 80% of the proteins (n = 1,392) and applied on the remaining 20% of the proteins (test set, n = 346). The plot shows only proteins from the test set. Ribosomal occupancies were taken from a reference dataset^56^ and log_10_-transformed. The proteome data were log_2_ transformed, centered, and scaled. (B) Gene Ontology (GO) slim term^37^ enrichment analysis of the top features selected by the model using a Fisher’s exact test (STAR Methods). (C) Half-lives are predicted with an elastic net model. The model was trained on 80% of the proteins (n = 1,398) and applied on the remaining 20% of the proteins (test set, n = 348). The plot only shows proteins from the test set. Half-lives were taken from a reference dataset^57^ and log_10_ transformed. The proteome data were log_2_ transformed, centered, and scaled. (D) The 15 most important KO strains in the regression model for half-lives. The KO strains are ranked by importance and scaled to have a maximum value of 100. (E) Abundance of ribosomal 60S subunit proteins in 10 KO strains that were selected as the most important feature for the prediction of protein half-life. Protein intensities are centered and log_2_-transformed. Significance for the comparison to the WT abundance levels (two-sided t test) is shown with asterisks (****p ≤ 0.0001; ***p ≤ 0.001; **p ≤ 0.01; *p ≤ 0.05; ^ns^p > 0.05). (F) Differential abundance of proteins with short (below median) and long (above median) half-lives (****p ≤ 0.0001, Wilcoxon signed-rank test). (G) Half-lives (in h, log_10_ transformed) are shown as boxplots for proteins that are predominantly decreased in abundance, increased in abundance, or change in both directions across the KO strains. Directionality was defined as ratios of increased and decreased abundance changes being >75% and <25% quantile for down and up, respectively. Significance (two-sided Wilcoxon signed-rank test with “no direction” as a reference) is shown with asterisks (****p value ≤ 0.0001; **p value ≤ 0.01). See also Figure S4.

We asked to what extent protein turnover and ribosome occupancy are important variables in determining differential protein expression. We used elastic net regression models^55^ and tested whether the proteomes can predict ribosome occupancy and protein half-life. Protein abundance values were used as predictor variables, and the protein half-lives or ribosome occupancies from reference datasets^56,57^ as response variables (see STAR Methods). We obtained high predictability in a hold-out test set (20% of proteins) and found that 60% of the variation in ribosome occupancies is explained by the regression model (R^2^ ~ 60%) (Figure 4A). Using the feature weights of the model, we assessed which gene deletions were most informative (Table S4). Processes related to RNA levels or transcription (“mRNA processing,” “DNA-templated transcription,” “RNA splicing,” and “transcription from RNA polymerase II promoter”) or protein degradation (“proteolysis involved in cellular protein catabolic process” and “protein modification by small protein conjugation”) were enriched (Figure 4B).

Next, we tested for the predictability of protein half-life, as obtained by metabolic labeling.^57^ As above, we constructed models using elastic net regression (STAR Methods) and obtained a high correlation of the measured and predicted half-lives in the hold-out set (Figure 4C). Here, the most informative gene deletions included *dur12Δ* (urea amidolyase), *sds24Δ* (a protein involved in cell separation), and *fun30Δ* (involved in chromatin remodeling) (Figure 4D; Table S5). Indeed, many proteins with short or long half-lives are differentially abundant in those strains (e.g., in *dur12Δ* long-lived proteins are increased, whereas in *fun30Δ*, long-lived proteins are decreased) (Figure S4A), indicating a changed equilibrium between translation and degradation. Although neither growth rate nor cell size is the main driver of those protein-half-life-dependent changes (Figure S4B), the translation machinery is significantly affected in most of those strains (Figure 4E).

Our results hence indicate that protein abundance, translation rate, and turnover are interdependent and act together in determining differential protein expression. Unexpectedly, our data revealed that proteins with a slow turnover (long half-life) are more likely to be differentially expressed (Figure 4F) and tend to be decreased in abundance (Figure 4G). For example, Sds24, Hsp26, and Pgm2, which are among the most long-lived proteins in yeast (half-lives > 130 h), are primarily downregulated (Figure S4C). We speculate that proteins with faster turnover rates are more easily buffered and may adapt better to genetic perturbations. Conversely, proteins with high ribosome occupancies are more likely to be differentially expressed (Figure S4D). Here, however, one needs to take some caution in the interpretation of that result. In contrast to half-life (Figure S4E), ribosome occupancy correlates with abundance,^58^ and the differential expression of a high-abundant protein is easier to detect.

### Disruption of protein complexes can lead to accelerated degradation of surplus subunits but can also lead to their induction when feedback loops are involved

**Figure 5:**
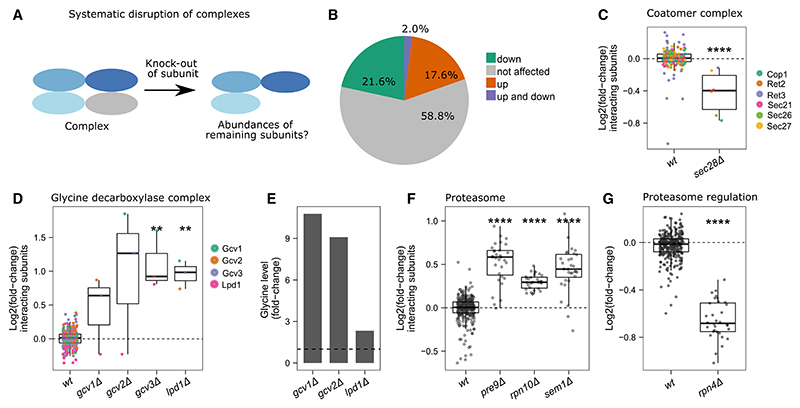
The response of protein complexes to genome-wide perturbation (A) Scheme: the response of complex subunits to the deletion of one subunit. (B) Fraction of complexes in which at least one deletion of a subunit induces a decrease (22%, green), increase (18%, orange), or in which some deletions induce increase and others decrease (2%, purple) of subunit abundances. The total number of considered complexes is 51 (STAR Methods). (C) Relative abundances of the coatomer complex subunits Cop1, Ret2, Ret3, Sec21, Sec26, and Sec27 are compared between s*ec28Δ* and WT samples. Data are centered and log_2_-transformed. (D) Relative abundances of the glycine decarboxylase complex subunits Gcv1, Gcv2, Gcv3, and Lpd1 are shown for the KOs of the glycine decarboxylase complex (*gcv1Δ*, *gcv2Δ*, *gcv3Δ*, and *lpd1Δ*) and WT samples. (E) Relative glycine abundances in glycine decarboxylase KOs (*gcv1Δ*, *gcv2Δ*, and *lpd1Δ*) are shown, as derived from a reference dataset.^15^ (F) The relative protein abundances of proteasome complex subunits in the viable KOs of the proteasome complex—*pre9Δ*, *rpn10Δ*, and *sem1Δ*—compared with their abundance levels in WT strains. Data are centered and log_2_-transformed. (G) The relative protein abundances of all measured proteasome subunits in *rpn4Δ* are compared with their WT abundance levels. Significance (two-sided Student’s t test with WT as a reference) is shown with asterisks (**** for p value ≤ 0.0001; *** for p value ≤ 0.001; ≤ for p value ≤ 0.01; * for p value ≤ 0.05).

It is assumed that many complex subunits are produced in super-stoichiometric amounts and that excess subunits (orphan subunits) are degraded.^49,51,59–61^ As our dataset allowed us to study the perturbation of all non-essential protein complex sub-units in a single study, we asked to which degree complex subunits are degraded on the deletion of a sub-unit (Figure 5A). In 22% of the studied complexes, at least one of the KOs caused a decrease in the other subunits (adjusted p value < 0.05, BH for multiple testing correction^62^) (Figure 5B). For example, the KO of the *SEC28* gene, where the gene product has a stabilizing function within the coatomer complex,^63^ decreases the abundance of its interacting subunits (Figure 5C). Other examples of subunits that lower the levels of interacting proteins are Paf1 in the PAF1 complex or Atp17 in the mitochondrial proton-transporting ATP synthase complex.

Notably, 18% of the studied complexes show an increased abundance in response to the deletion of at least one subunit (Figure 5B). In the search for an explanation, we noted complexes that are regulated by a known transcriptional or metabolic feedback loop. For example, subunits of the glycine decarboxylase complex, which regulates one-carbon metabolism via methylene tetrahydrofolate,^64^ are increased when glycine levels are high.^65^ Indeed, the deletion of a subunit of the glycine decarboxylase complex (*gcv1Δ, gcv2Δ*) increased glycine levels (Figures 5D and 5E, re-processed data^15^). Another example is the proteasome complex (Figure 5F), which is regulated by the short-lived transcription factor Rpn4 via a negative feedback loop to maintain proteasome levels under cellular stress.^66–68^ Indeed, although the deletion of subunits resulted in an increased abundance of the other complex members, the deletion of this transcription factor resulted in the downregulation of the proteasome complex (Figure 5G).

### The impact of genetic perturbations on the functional global proteome

**Figure 6:**
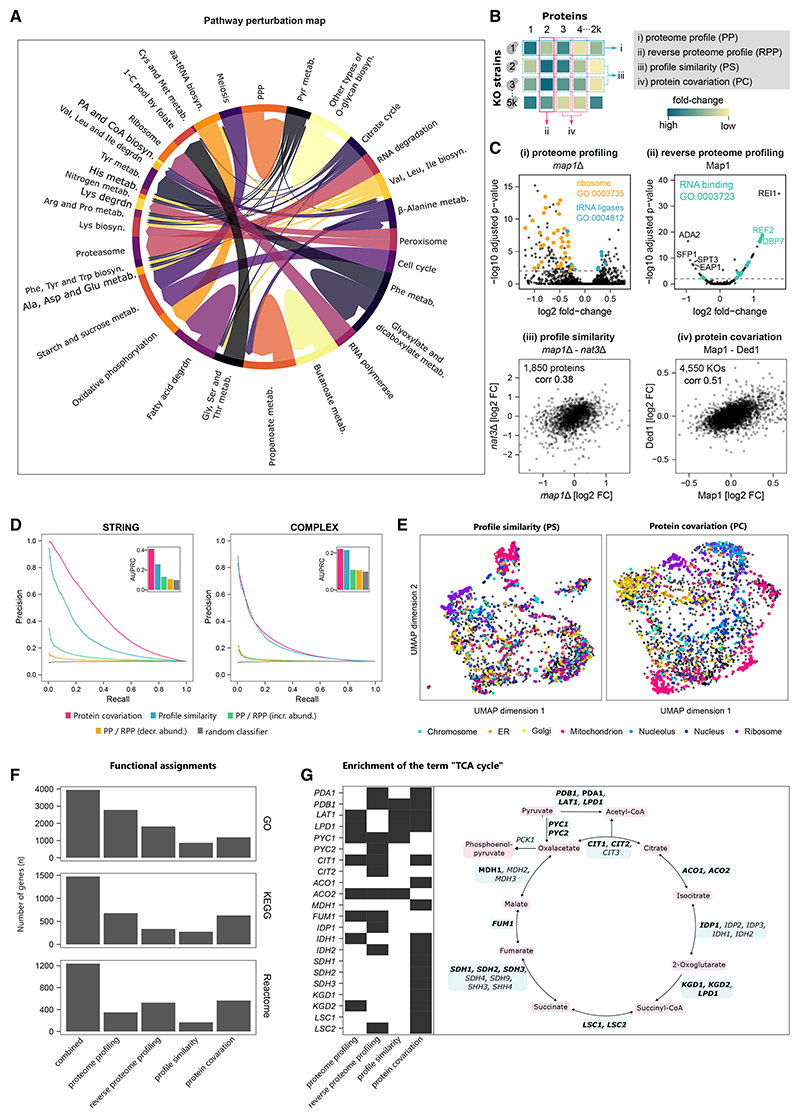
Annotating gene functions using functional proteomics (A) Map connecting genetic perturbations to the corresponding proteome response. Genes are grouped by KEGG pathway,^69,70^ arrows point from perturbed toward affected pathways (STAR Methods). PPP, pentose phosphate pathway; metab., metabolism; biosyn., biosynthesis; degrdn, degradation; 1-C, one carbon; PA, pantothenate; aa, aminoacyl; Pyr, pyruvate; amino acids indicated by standard three-letter code. (B) The four functional annotation strategies supported by this dataset. (C) The *MAP1* gene exemplifies the complementary nature of these proteome annotation strategies. (Ci and Cii) Volcano plots of proteome profile and reverse proteome profile of the *map1*Δ strain and Map1 protein, respectively. Dashed lines indicate significant changes (adjusted p value < 0.01). (Ciii) Protein fold-changes (FC) measured in the *map1Δ* strain are similar to those in the *nat3Δ* strain (Spearman correlation = 0.38). (Civ) Abundance changes of *Map1* and *Ded1* proteins are correlated across all strains (Spearman correlation = 0.51). (D) Precision-recall analyses showing that profile similarities (PSs) and protein covariation (PC) capture gene function very well. In addition, protein-KO pairs were ranked by the protein fold-change in the KO, showing that the extent of upregulation (PP/RPP [incr. abundance]) or downregulation (PP/RPP [decr. abundance]) is a relatively poor indicator of shared protein/KO function. Performance was assessed using two gold standards for shared protein function, STRING^73^ (left) and COMPLEAT protein complexes^74^ (right). Only responsive KOs were considered for profile similarity analysis. See STAR Methods for details. (E) Functional maps created using uniform manifold approximation and projection (UMAP), grouping KO strains by profile similarity (left) and proteins by covariation (right). Subcellular compartment annotation shows that both approaches capture subcellular organization. (F) Number of genes that could be associated with at least one GO term, KEGG pathway or Reactome pathway by over-representation analysis. For PPs, the enrichment was performed on the differentially expressed proteins in each strain and for RPPs the KOs in which the respective protein was differentially expressed. For PS and PC, we considered the highest-scoring 1% of associations in the networks. Functional enrichment was considered significant for p < 0.01 (topology-weighted topGO analysis) or BH-adjusted p < 0.01 (KEGG/Reactome Fisher’s exact test, STAR Methods). (G) Functional annotations capture known interactions within the TCA cycle. The KEGG term “TCA cycle” was enriched in 22 TCA cycle genes by at least one of the annotation methods, 6 by two methods, and 6 by three. See also Figure S5.

To globally study the functional consequences of genetic perturbations on the proteome, we grouped the gene-deletion strains on a pathway-by-pathway basis using the KEGG pathway annotation.^69,70^ Then, we characterized the proteomic responses by gene-set analysis (Figure 6A). The analysis revealed that the proteome captures global relationships between perturbed and responding pathways. The most common responses to any genetic perturbation were enriched for metabolism, with amino-acid and nucleotide metabolism being among the most frequently responding gene sets (Figure 6A). This result reflects that the metabolic network is the largest interconnected biological system^71^ and known to be responsive to the general physiological changes.^15^ For example, KOs related to pyruvate metabolism show proteome responses in various amino-acid metabolic and biosynthetic pathways (i.e., *His, Arg, Pro, Lys, Phe, Tyr, Trp, Ala, Asp, Glu, Gly, Ser*, and *Thr*). We further found that perturbations of the peroxisome result in differential abundance in lysine biosynthesis and lysine degradation (Figure 6A), reflecting that lysine metabolism is connected to peroxisome deficiency.^72^

Another interesting result indicated that perturbing RNA degradation induces the proteasome (Figures 6A, S5A, and S5B). An increase in RNA levels could hence be compensated through more protein degradation. For example, *mot2Δ* or KOs of the LSM complex subunits (*lsm1Δ*, *lsm6Δ*, and *lsm7Δ*) have increased levels of the proteasome (Figure S5B).

### Using functional proteomics to annotate gene function

Although 2,913 yeast genes are well annotated in the sense that they reach the highest UniProt annotation score (5 of 5) and have a median of 103 publications each, there are also 468 yeast genes that have the lowest score (1 of 5) and are mentioned in a median of only 4 publications (Figures S5C and S5D). We report four successful and complementary strategies of annotating proteins through functional proteomics, of which three are specifically facilitated by the large-scale combination of functional genomics and proteomics (Figure 6B): (1) interpretation of a KO strain’s PP, (2) interpretation of a protein’s response across KOs (reverse proteome profile [RPP]), (3) a “guilt-by-association” approach, grouping KOs with similar PPs together (profile similarity [PS]), and (4) grouping proteins based on their co-expression across KOs (protein covariation [PC]).

Associating KO strains by PS was previously successful for annotating gene function using transcriptomics^14^ and metabolomics.^15^ However, the scale of our proteomics dataset presented a challenge for this annotation strategy, as the distance metrics struggle to calculate meaningful similarities in high-dimensional data.^75^ We therefore devised a feature-selection strategy, based on the observation that proteins that are informative for predicting growth rates are also informative for assessing KO strain similarity. Selecting 185 (10%) proteins in this manner and applying a topological overlap measure^76^ substantially improved the detection of functionally related genes (Figures S6A–S6E; STAR Methods). We also observed that PPs of 2,290 “responsive” KO strains (strains with more differentially expressed proteins than the median strain) could be compared particularly well (Figure S6F). We therefore focused our subsequent analysis of PPs on the responsive strains. Feature selection also proved beneficial for PC analysis. For this, we ranked KO strains by the number of differentially expressed proteins. We found that selecting the 10% most responsive KO strains (467 of 4,675) significantly improved the PC analysis (Figures S6G–S6I).

Annotating methionine aminopeptidase 1 (Map1) illustrates the complementary nature of the four approaches (Figure 6C). Map1 co-translationally removes the N-terminal methionine from nascent proteins. The PP of *map1*Δ reveals 205 differentially abundant proteins, enriched for ribosomal proteins and tRNA ligases (Figure 6Ci). By contrast, RPP revealed that the Map1 protein is upregulated upon the deletion of ribosome biogenesis factors *rei1*Δ and *dbp7*Δ and more generally in KOs of RNA-binding proteins. Map1 protein levels are reduced in the *sfp1*Δ strain, a transcription factor that regulates ribosome biogenesis gene expression, and upon the deletion of subunits of the SAGA transcriptional coactivator complex (*ada2*Δ, *spt3*Δ, and *gnc5*Δ) (Figure 6Cii). Third, clustering the profiles by similarity revealed a close relationship between *map1*Δ and *nat3*Δ. Indeed, Nat3 catalyzes the acetylation of N-terminal methionines of nascent proteins (Figure 6Ciii). Finally, exploring proteins with similar response patterns (PC) across KO strains reveals that Map1 protein strongly correlates with the expression of Ded1, an RNA helicase involved in translation initiation (Figure 6Civ).

Next, we assessed the global performance of the annotation strategies. We ranked KO-protein pairs by the fold-change and subjected them to precision-recall (PR) analysis, using two different gold standards as reference: functional associations mapped by STRING^73^ and interactions between protein complex subunits mapped by COMPLEAT.^74^ Although the extent of upregulation of a protein is moderately indicative of a shared function with the deleted gene, the extent of downregulation is not (Figure 6D). We then tested how well KO-KO and protein-protein similarity scores recapitulate the known interactions. Both protein PSs and PC detect these associations well (Figure 6D). We visualized the overall gene-gene (or protein-protein) associations using uniform manifold approximation and projection (UMAP) analysis.^77^ We created two maps in which similar KOs (or proteins) are grouped together (Figure 6E). Although our methods do not directly measure physical interactions, grouping proteins by functional similarity means that both maps partially reflect the subcellular organization of the cell (Figure 6E).

In addition to these pairwise associations, we also tested whether the groups of linked KOs or proteins were enriched for biological function terms (Figure 6F; STAR Methods). We found 2,782, 678, and 349 PPs enriched for at least one GO term, KEGG, or Reactome pathway, respectively (Figure 6F). The annotations are complementary as the strategies together annotate more genes/proteins than each of the individual scores alone. In total, 3,947, 1,474, and 1,238 genes/proteins could be assigned at least one GO, KEGG, or Reactome term (Figure 6F). We then focused this analysis on the 1,086 most under-studied yeast genes (Figures S5C and S5D) and found that 501 (of the 849 covered by our analysis) could be associated with at least one functional term (Figure S5E).

To illustrate the combined power of our approaches, we inspected the interactions reported for the enzymes of a metabolic pathway, the tricarboxylic acid (TCA) cycle. From the 33 PPs, RPPs, PSs, and PCs of genes belonging to the corresponding KEGG term,^69,70^ 22 have significant enrichments of this term (Figure 6G). For example, the pyruvate carboxylase (*pyc1Δ*) that converts pyruvate to oxaloacetate has a similar profile with *pdb1Δ*, *aco2Δ*, *lpd1Δ*, *lat1Δ*, and *idh1Δ* (Figure S5G). Interestingly, the PC analysis highlights different associations and found covariations of *Pyc1* with *Pyc2, Idp1, Idh2*, and *Cit2* (Figure S5G). Complementary associations for *pyc1Δ* were also observed by PP analysis (*Idp1, Cit1, Cit2, Fum1, Pdb1, Pda1*, and *Aco2*) and RPP analysis (*idh1Δ*, *aco2Δ*, *fum1Δ*, *cit1Δ*, and *lat1Δ*) (Figures S5H and S5I). Furthermore, our approaches are complementary to genetic interactions^78^ where significant enrichments were found for 13 of the 33 TCA-cycle-related genes (Figure S5J). The covariation analysis of the TCA cycle enzymes highlights another interesting observation: the paralogs *Cit1* (mitochondrial citrate synthase) and *Cit2* are found in 2 different clusters (Figure S5G), reflecting that they diverged functionally. Although *Cit1* covaries with *Fum1, Kgd1 Sdh1, Sdh2, Mdh1, Lsc1*, and *Lat1*, its paralog *Cit2* covaries with *Pyc1, Pyc2, Idh1, Idh2*, and *Idp1* (Figure S5G).

#### Functional proteomics provides orthogonal information to functional genomics

We compared the highest-scoring 1% of the pairwise associations found by PS (n = 26,210 KO pairs, Table S6) and PC analysis (n = 26,255 protein pairs, Table S7). They connect a subset of 1,284 KOs and 1,396 proteins, respectively. Some of these genes are linked to fewer than five other genes, others to more than 100 genes (Figure S7A; STAR Methods). Interestingly, there is very little overlap between these top 1% pairwise associations (Figure S7B). This indicates that proteome profiling and KO profiling not only detect different genes (Figure S5F) but indeed different types of associations. Connecting KOs by proteome PS preferentially captures genetic over physical interactions and associations that were previously detected by literature text mining (Figures S7C and S7D). By contrast, PC analysis captures physical interactions better than genetic interactions and agrees best with associations previously found through mRNA co-expression (Figures S7C and S7D). Together, these data suggest that proteome and KO profiling provide two complementary dimensions for gene-function characterization.

One of the most successful genome-scale approaches of functional genomics is SGAs that detect genetic interactions.^38,78^ To understand how our approach compares to genetic interactions in associating genes to function, we divided associations based on whether they connected essential or non-essential genes and whether they gave rise to positive or negative genetic interactions (Figure S7E). Although KO studies do not cover essential genes, PC does (Figures S5F and S7E). Intriguingly, PR analysis reveals that PSs are better suited for detecting associations between KOs that have positive genetic interactions than those that have negative ones. In fact, for positive associations, PS outperforms the original genetic interaction scores, which more precisely identify functional links between negatively interacting genes (Figure S7E). The PR performance of PC is consistently strong and not affected by gene essentiality or the nature of the genetic interaction (Figure S7E).

### Exploring functional relationships within the yeast proteome

**Figure 7:**
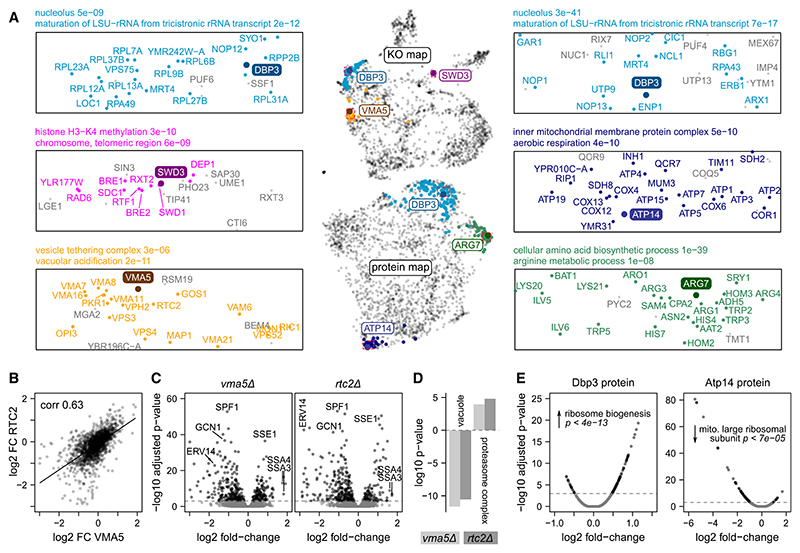
Exploring functional relationships in a proteomic map of genome-scale perturbation (A) Proximity in the UMAPs of KO strains and proteins reflects functional similarity. Three KOs (top map/left panel) and three proteins (bottom map/right panel) are shown as examples. KOs/proteins that are strongly linked to the example gene (within 1% highest-scoring associations, STAR Methods) are highlighted in color. Selected GO terms enriched among these groups are indicated (enrichment p value from Fisher’s exact test). (B) Protein fold-changes (FC) of two KOs that are near each other in the UMAP (*vma5Δ* and *rtc2Δ*, bottom left in A) are strongly correlated (biweight midcorrelation coefficient = 0.63). (C) Volcano plots of the PPs of the same KOs, revealing many overlapping differentially expressed proteins, a few of which are labeled. (D) GO term enrichment for differentially expressed proteins using a Mann-Whitney U test, revealing that vacuolar proteins are depleted in both KOs, whereas the proteasome is enriched. (E) Abundance changes of two example proteins, Dbp3 and Atp14, across KO strains are shown using volcano plots (RPP). Same GO enrichment analysis as in (D), showing that, e.g., Dbp3 abundance is increased in KO strains related to “ribosome biogenesis.”

To gain more insights into the functional relationships detected, we explored several profiles in more detail (Figure 7). Dbp3 is an RNA helicase involved in pre-rRNA processing,^79^ which our dataset contains both as a KO and as a quantified protein. Dbp3 locates to the nucleolar region of both the KO and protein maps and is linked to other rRNA maturation and ribosome biogenesis factors at both levels (Figures 7A and 7E). However, proteome PS and PC detect a different subset of ribosome biogenesis factors. Similar functional relationships can be explored for all genes that were captured either at KO or protein level (e.g., *SWD3*, Atp14, and Arg7; Figures 7A and 7E).

Furthermore, proteomes offer detailed insights into why two gene deletions can be similar in their biological impact. For example, the *VMA5* gene encodes a subunit of the vacuolar membrane H^+^-ATPase.^80^ In the KO similarity map, *vma5Δ* clusters together with many other genes with vacuolar functions, including genes encoding other H^+^-ATPase subunits (Figure 7A). One of its associated KOs is the putative vacuolar membrane transporter *RTC2*. The PPs of the *vma5Δ* and *rtc2Δ* strains are strongly correlated (Figure 7B), and they share a number of differentially expressed proteins, such as an increase in heat-shock proteins Ssa3, Ssa4, and Sse1 (Figure 7C). GO analysis reveals that, in both KOs, the abundance of vacuolar proteins is decreased, and the abundance of the proteasome is increased (Figure 7D). Such insights facilitate hypothesis generation for future mechanistic gene-function studies. For example, it is possible that vacuolar defects in the *vma5Δ*, *rtc2Δ*, and related KO strains lead to an accumulation of damaged proteins, inducing the unfolded protein response that involves heat-shock factors and the proteasome.

## Discussion

Genome-scale profiling of loss-of-function mutants has been successfully used to map biological networks and gene function.^6^ Functional genomic profiling has been extensively applied at the phenotypic level. The Yeast Phenome database (www.yeastphenome.org) lists phenotypes of single-gene deletion strains across 7,536 experimental conditions.^81^ Our study provides a significant amount of molecular data to help interpret the detected phenotypes. Moreover, for associating functional terms to genes, the proteome is complementary to these approaches and provides added value to other “functional omic” screens, as neither transcriptome nor metabolome captures the post-transcriptional regulation of protein expression. For instance, we herein identify protein complexes for which the degradation of surplus subunits is induced when a gene encoding a complex subunit is disrupted. Moreover, our dataset puts such findings into context. We show that 20% of the studied complexes behave differently and are increased upon the deletion of one subunit. Our data indicate that, in these cases, feedback control mechanisms could be involved.

Moreover, functional proteomics generates insights into the general principles that govern protein expression. On the one hand, we confirm and quantify the paradigm that proteomic responses are driven by the function of the deleted protein. Paralogs and proteins connected in genetic, metabolic, evolutionary, or protein-protein interaction networks have a higher likelihood of responding to the deletion of the connected gene. At the same time, however, our dataset also shows that large fractions of protein abundance changes are explained by general biological properties that affect the proteome as a whole. These properties include the location of a protein-coding gene on a potentially aneuploid chromosome, growth rate, translation rate, and protein turnover.

Eventually, our study demonstrates added value for gene annotation through the systematic generation and analysis of proteomes. Through RPP, which identifies the genetic perturbations that trigger an expression change in a particular protein, and two guilt-by-association approaches^82,83^ that infer gene function through proteome PS and proteins with similar expression patterns (PC), respectively, we show that annotation strategies capture known and unknown functional associations. Thus, the combination of multiple omic technologies with complementary strengths and biases could become a paradigm for providing accurate and comprehensive data-driven gene-function annotation. This is especially relevant for future studies addressing the problem of understudied proteins, not only in model organisms but also in a wide range of species and genetic backgrounds.

### Limitations of the study

Although the yeast genome-scale KO collection is considered an excellent genetic library and has been used in a large number of studies,^6^ it contains a low number of false negatives and false positives and a subset of strains contain compensatory mutations.^6,84,85^ We have estimated from our data that more than 90% of the KOs have the correct gene deleted (Figures S1D–S1F) and designed our analyses to minimize the effects. Nevertheless, some individual results from our dataset demand replication in subsequent, focused studies.

Moreover, we chose a minimal medium and a prototrophic background because research from ourselves and others has shown that rich media compositions result in the feedback inhibition of many metabolic pathways because cells uptake instead of synthesize metabolites.^15,86^ However, the proteome response is dependent on both the background and condition. We measured and compared a subset of the KOs in a related background and found diverging proteome responses for some genes (Figures S1J–S1L). Hence, additional proteomic analyses will be required in the future and not all yeast studies are directly comparable because of genetic background, the use of auxotrophs, and differing media.

Furthermore, our study reports a single proteome per KO strain, and the reported fold-changes are based on relative quantification. Although we show for strains with chromosomal duplications that our technology overall captures expected protein changes (Figures 2E, 3F, 3I, and 3J) and that the use of large numbers of wild-type replicates increases the detectability of differential protein abundances (Figures S1G–S1I), we cannot exclude discrepancies for individual proteins. However, we and many others in the field are active in developing next-generation proteomic technologies that will drive larger studies with absolute quantitative measurements in the future.

## Star⋆Methods

### Key Resources Table

**Table:** 

REAGENT or RESOURCE	SOURCE	IDENTIFIER
Chemicals, peptides, and recombinant proteins		
Water, Optima, LC-MS Grade, Optima, Fisher Chemical	Fisher Scientific	Cat#10509404; CAS: 7732-18-5
Acetonitrile, Optima, LC-MS Grade, Fisher Chemical	Fisher Scientific	Cat#10489553; CAS: 75-05-8
Thermo Scientific Pierce Formic Acid, LC-MS Grade	Fisher Scientific	Cat#13454279; CAS: 64-18-6
Methanol, Optima LC/MS Grade, Thermo Scientific	Fisher Scientific	Cat#10767665; CAS: 67-56-1
Yeast nitrogen base without amino acids	Sigma-Aldrich	Cat#Y0262
D-(+)-Glucose	Sigma-Aldrich	Cat#G7021; CAS: 50-99-7
DL-Dithiothreitol (BioUltra, for molecular biology, >=99.5%)	Sigma Aldrich	Cat#43815; CAS: 3483-12-3
Iodoacetamide (BioUltra)	Sigma Aldrich	Cat#I1149; CAS: 144-48-9
solid-glass beads (borosilicate, diam 4 mm)	Sigma Aldrich	Cat#Z143936
ammonium bicarbonate (eluent additive for LC-MS)	Sigma Aldrich	Cat#40867; CAS: 1066-33-7
Urea (puriss. P.a., ACS reagent, reag. Ph. Eur., >=99.5%)	Honeywell Research Chemicals	Cat#33247H; CAS: 57-13-6
Acetic acid (Eluent additive for LC-MS)	Honeywell Research Chemicals	Cat#49199; CAS: 64-19-7
Trypsin (Sequence grade)	Promega	Cat#V5117
iRT peptides	Biognosys	Cat#Ki-3002-b
Deposited data		
Raw proteome data	This study	ProteomeXchange: PXD036062
Processed proteome data	This study	Mendeley Data:http://doi.org/10.17632/w8jtmnszd9.1
Growth rates	This study	Mendeley Data:http://doi.org/10.17632/w8jtmnszd9.1
Yeast reference proteome databases	Uniprot	https://www.uniprot.org
Ribosomal profiling data	McManus et al.^56^	http://doi.org/10.1101/gr.164996.113
Protein turnover rates	Martin-Perez and Vill^57^	https://doi.org/10.1016/j.cels.2017.08.008
Gene networks	Kim et al.^34^	https://www.inetbio.org/yeastnet/
Complex data	Medal et al.^87–89^	https://www.ebi.ac.uk/complexportal/
Glycine concentrations	Mulleder et al.^15^	http://doi.org/10.1016/j.cell.2016.09.007
Full GO term annotation	Gene Ontology Consortium	http://current.geneontology.org/products/ pages/downloads.html
GO slim terms	Cherry et al.^37^	https://www.yeastgenome.org/
Colony size	Cherry et al.^37^	https://www.yeastgenome.org/
Reactome	Gillespie et al.^90^	https://reactome.org/
KEGG	Kanehisa and Goto^69^; Kanehisa^70^	https://www.genome.jp/kegg/
BioGRID	Stark et al.^91^	https://thebiogrid.org
Yeast phenotype data (e.g. gene essentiality)	Cherry^92^	http://sgd-archive.yeastgenome.org/curation/ literature/phenotype_data.tab
Protein abundances for all yeast proteins (meta-analysis)	Ho et al.^93^	https://doi.org/10.1016/j.cels.2017.12.004
List of uncharacterised yeast genes	YeastMine	https://yeastmine.yeastgenome.org/yeastmine/bagDetails.do?scope=all&bagName=Uncharacterized_ORFs
Citations mapped to yeast genes	Saccharomyces Genome Database	http://sgd-archive.yeastgenome.org/curation/ literature/gene_literature.tab
S. cerevisiae Ohnologs	Yeast gene order browser^35^	http://ygob.ucd.ie/
Classification of duplicates	Kuepfer et al.^36^	http://doi.org/10.1101/gr.3992505
COMPLEAT	Vinayagam et al.^74^	http://www.flyrnai.org/compleat
Genetic interactions	Costanzo et al.^78^	https://thecellmap.org/costanzo2016/
STRING	Szklarczyk et al.^73^	https://string-db.org
Experimental models: Organisms/strains		
Prototrophic Saccharomyces cerevisiae deletion collection (MATa, restored prototrophy)	Winzler et al.^5^; Mülleder et al.^94^	http://www.euroscarf.de/
Software and algorithms		
Proteomics data analysis via Deep Neural Networks, DIA-NN	Demichev et al.^28^	https://github.com/vdemichev/DiaNN
DIA-NN R package	Demichev et al.^28^	https://github.com/vdemichev/diann-rpackage
R Statistical Computing Software	The R Foundation	https://www.r-project.org/
tidyverse	Wickham et al.^95^	https://cran.r-project.org/web/packages/tidyverse/
treeClust R package	Buttrey and Whitaker^96^	https://CRAN.R-project.org/package=treeClust
caret R package for regression modeling	Kuhn et al.^97^	https://CRAN.R-project.org/package=caret
Impute R package	Hastie et al.^98^	https://bioconductor.org/packages/impute/
randomForest R package	Liaw and Wiener^99^	https://CRAN.R-project.org/package=randomForest
WGCNA R package	Zhang and Horvath^100^; Langfelder and Horvath^101^	https://CRAN.R-project.org/package=WGCNA
PRROC R package	Grau et al.^102^	https://CRAN.R-project.org/package=PRROC
ComplexHeatmap R package	Guet al.^103^	https://bioconductor.org/packages/ComplexHeatmap/
Circlize R package	Guet al.^104^	https://CRAN.R-project.org/package=circlize
Piano R package	Väremo et al.^105^	https://github.com/varemo/piano
clusterProfiler	Väremo et al.^105^	https://bioconductor.org/packages/clusterProfiler/
topGO R package	Alexa and Rahnenfuhrer^106^	https://bioconductor.org/packages/topGO/
limma R package	Ritchie et al.^107^	https://bioconductor.org/packages/limma/
Other		
96-Well MACROSpin C18, 50–450 μL	The Nest Group	Cat#SNS SS18VL
HSS T3 column (150 mm x 300 mm, 1.8 μm particles)	Waters	Cat#186009249
Breathe-Easy sealing membrane for multiwell plates	Sigma Aldrich	Cat#Z763624
Adhesive PCR plate foil	Thermo Scientific	Cat#AB0626
ABgene storage plates	Thermo Scientific	Cat#AB-0661
Glass beads, acid-washed (425-600 μm)	Sigma Aldrich	Cat#G8772
Cap mats	Spex	Cat#2201
Corning multiwell plates, plate lids and sealing mats	Sigma Aldrich	Cat#CLS3098
96-well Sample Collection plate (700 μl round well)	Waters	Cat#186005837
Pierce Quantitative Peptide Assays & Standards	Thermo Scientific	Cat#23290

### Resource Availability

#### Lead contact

Further information and requests for resources and reagents should be directed to and will be fulfilled by the lead contact, Markus Ralser (markus.ralser@charite.de).

#### Materials availability

Requests for reagents should be directed to and will be fulfilled by the lead contact.

### Experimental Model and Subject Details

#### Strains and library layout

We measured proteomes for all strains of *Saccharomyces cerevisiae* (S288c) haploid (MATa) deletion collection^5^ with restored prototrophy^94^ that could be cultivated without major growth defect in minimal dextrose medium. To conduct the study, the single knock-out strains were arranged on 96-well plates. A blank was introduced in each plate in a different position as a plate identifier. This moving footprint starts at H12 and runs backwards (skipping control positions). The control strain (388 replicates) is the complemented *his3Δ* deletion strain, haploid from a BY4741 prototrophic deletion collection. This control strain was introduced in 7 positions on each plate: A11, B8, C5, D2, F11, G8, H5. Plates 56 and 57 contain additional controls.

#### Culture

The yeast strains were grown in batches of 12 96-well plates. In order to reduce batch effects, the media for all batches were prepared at once, pre-filled into 96-well plates, and stored at –80°C until the day of the experiment. Further, a 5x synthetic minimal (SM) medium stock solution was prepared and stored at –80°C and used for the agar plates, which were prepared fresh on the day of the experiment. All media were filtered (0.22 μm filter, GP Millipore Express Plus membrane) and the plates as well as the beads were autoclaved before usage. All pipetting was done with a Biomek NX^P^ liquid-handling robot (Beckmann) and yeast cells were pinned with a pinning robot (Rotor, Singer Instruments).

The yeast strains were grown as previously published^15^ with slight modifications. The thawed stock cultures were spotted with the pinning robot onto SM agar medium (6.7 g/l yeast nitrogen base without amino acids, 2% glucose, 2% agar) and incubated at 30°C for 47–49 hours. Subsequently, these cells were used for inoculation in 200 μl SM liquid medium in 96-well plates and incubated at 30°C. After 19.75 hours, 160 μl culture was transferred to a deep-well plate (ABgene storage plates) pre-filled with 1,440 μl SM liquid medium (1/10 dilution) and with one solid-glass bead (borosilicate) per well. The plates were sealed with a membrane (Breathe-Easy sealing membrane for multiwell plates) and incubated for 8 hours at 30°C with 1,000 rpm mixing (Heidolph Titramax incubator). Sub-sequently, the culture was transferred into a fresh 96-well plate (Eppendorf, 10052143) and spun down at 4,000 rpm (Eppendorf Centrifuge 5810R). The supernatant was removed and the plate was sealed with aluminium foil (adhesive PCR plate foil) as well as a plastic lid (CLS3098) before being frozen and stored at –80°C until further processing.

For the comparison with the SGA background, strains were cultivated as described above, except that 80 μl of pre-culture were transferred into deep-well plates pre-filled with 1,550 μl of SM liquid medium (1/20 dilution).

## Method Details

### Proteomic sample preparation

The protein extraction and digestion were conducted in batches of 4 plates (384 samples). In order to reduce batch effects, stock solutions (120 mM iodoacetamide, 55 mM DL-dithiothreitol, 9 μl 0.1 mg/ml trypsin, 2 μl 4x iRT) were prepared at once and stored at –80°C. Other stock solutions (7 M urea, 0.1 M ammonium bicarbonate, 10% formic acid) were stored at 4°C. All pipetting was done with a Biomek NX^P^ liquid-handling robot (Beckmann), shaking was done with a Thermomixer C (Eppendorf) after each step, and for incubation a IPP55 incubator (Memmert) was used.

200 μl 7 M urea / 100 mM ammonium bicarbonate and glass beads (~100 mg/well, 425–600 μm) were added to the frozen pellet. Subsequently, the plates were sealed (Cap mats, (Spex) 2201) and lysed using a Geno/Grinder (Spex) bead beater for 5 min at 1,500 rpm. After 1-min centrifugation at 4,000 rpm, 20 μl 55 mM DL-dithiothreitol were added (final concentration 5 mM), mixed, and the samples were incubated for 1 h at 30°C. Subsequently, 20 μl 120 mM iodoacetamide were added (final concentration 10 mM) and incubated for 30 min in the dark at room temperature. 1 ml 100 mM ammonium bicarbonate was added, centrifuged for 3 min at 4,000 rpm, then 230 μl were transferred to prefilled trypsin plates. After incubation of the samples for 17 h at 37°C, 24 μl 10% formic acid were added. The digestion mixtures were cleaned up using C18 96-well plates. For solid-phase extraction, 1 min of centrifugation at the described speeds (Centrifuge 5810R (Eppendorf)) was used to push the liquids through the stationary phase and the liquid handler was used to pipette the liquids onto the material. The plates were conditioned with methanol (200 μl, centrifuged at 50 *g*), washed twice with 50% ACN (200 μl, centrifuged at 50 *g*, then the flow-through discarded), equilibrated three times with 3% ACN, 0.1% FA (200 μl, centrifuged at 50 *g*, 80 *g*, 100 *g*, respectively, then the flow-through discarded). 200 μl of digested samples were then loaded (centrifuged at 100 *g*) and washed three times with 3% ACN, 0.1% FA (200 μl, centrifuged at 100 *g*). After the last washing step, the plates were centrifuged another time at 180 *g* before the peptides were eluted in 3 steps (twice with 120 μl and once with 130 μl 50% ACN, 180 *g*) into a collection plate (1.1 ml, square well, V-bottom). Collected material was completely dried in a vacuum concentrator (Concentrator Plus (Eppendorf)) and redissolved in 40 μl 3% ACN, 0.1% formic acid before being transferred into a 96-well plate (700 μl round, Waters, 186005837) prefilled with iRT peptides (2 μl, 4x diluted). QC samples for repeat injections were prepared by pooling digested and cleaned-up samples from 4 different 96-well plates.

2 μl of each sample were loaded onto ‘Lunatic’ microfluidic 96-well plates (Unchained Labs). Peptide concentrations were measured with the Lunatic instrument (Unchained Labs). Protein concentrations were calculated from the absorbance value at 280 nm and the protein-specific extinction coefficient.

For the comparison with the SGA background, samples were processed as described above, with the following adaptations: after reduction and alkylation, samples were diluted using 460 μl of 0.1 M ammonium bicarbonate, and 500 μl of this mixture were digested using 2 μg trypsin/LysC; the digest was stopped by adding 25 μl 25% formic acid; dried peptides were dissolved in 70 μl 0.1% formic acid. As a technical control for MS measurements, 10 μl of each sample were pooled together and the peptide concentration of this pool was determined using a fluorimetric peptide assay kit (Thermo Scientific, 23290). Peptide concentrations of the samples before injection were estimated based on the optical densities of the samples at harvest and the peptide pool concentration.

### Deletion mutants in the SGA strain background

We constructed a diploid background by mating the BY4741 strain (*MATa ura3Δ0 leu2Δ0 his3Δ1 met15Δ0*) with Y7092, a starting strain that carries markers for SGA selection (*MATa can1Δ::STE2pr-Sp_his5 lyp1Δ* *ura3Δ0 leu2Δ0 his3Δ1 met15Δ0*). The resulting diploid is compatible with the standard sporulation/haploid selection procedure used in SGA.^38^ We selected 29 genes that have broad proteome profiles but wild-type-like growth rates in the prototrophic deletion collection, and performed gene deletion in the SGA-compatible diploid background using plasmid constructs for direct homologous gene deletion in diploid isolates based on CRISPR-Cas9 as described previously.^108^ Briefly, a fragment carrying the *natMX* marker bordered by ~200 bp of sequences homologous up- and downstream of the targeted gene was cloned onto a plasmid backbone containing spCas9, a guide RNA, the *URA3* marker, the yeast *CEN6* sequence fused to an autonomous replication sequence, as well as an ampicillin resistance marker and an *E. coli* replication origin site from the standard pBluescript SK II (+). The 29 plasmids were individually transformed into the SGA-compatible diploid background on SD-Ura+NAT medium. The transformants were subsequently transferred onto YP galactose 2% to induce the expression of the CRISPR-Cas9 system, where site-specific double-strand breaks were induced to favour the gene deletion by homologous recombination. Deletion mutants were then selected on SC+5-FOA+NAT medium for integration of the deletion fragment as well as the loss of the plasmid. After this procedure, the diploid starting strain will either carry a homozygous or heterozygous deletion at the targeted locus. To mimic the double deletion mutant selection following the SGA procedure, diploid deletion mutants were carried through the SGA selection steps, namely sporulation on Spo medium (1% potassium acetate + 0.1% glucose), then on SC+canavanine+thialysine+NAT. The resulting deletion mutants carry the same genotype as SGA double mutants (*MATa,yfg1Δ::NAT can1Δ::STE2pr-Sp_his5 lyp1Δ* *ura3Δ0 leu2Δ0 his3Δ1 met15Δ0*).

### Liquid chromatography–mass spectrometry

The digested peptides were analysed on a nanoAcquity (Waters) running as microflow LC (5 μl/min), coupled to a TripleTOF 6600 (SCIEX). 2 μg of the yeast digest (injection volume was adjusted for each sample based on the measured peptide concentration) were injected and the peptides were separated in a 19-min nonlinear gradient (Table S1) ramping from 3% B to 40% B (solvent A: 1% acetonitrile/0.1% formic acid; solvent B: acetonitrile/0.1% formic acid). A HSS T3 column (Waters, 150 mm × 300 μm, 1.8 μm particles) was used with a column temperature of 35°C. The DIA acquisition method consisted of an MS1 scan from m/z 400 to 1250 (50 ms accumulation time) and 40 MS2 scans (35 ms accumulation time) with variable precursor isolation width covering the mass range from m/z 400 to 1250 (Table S2). Rolling collision energy (default slope and intercept) with a collision energy spread of 15 V was used. A DuoSpray ion source was used with ion source gas 1 (nebuliser gas), ion source gas 2 (heater gas), and curtain gas set to 15 psi, 20 psi, and 25 psi. The source temperature was set to 0°C and the ion-spray voltage to 5,500 V. The measurements were conducted within a period of 12 months and on 2 different platforms with identical setups.

For the comparison with the SGA background, wild-type and KO strains were analysed on a UltiMate 3000 RSL (Thermo) coupled to a TimsTOF PRO (Bruker) mass spectrometer. Peptides were separated on the same column (Waters ACQUITY UPLC HSS T3 1.8 μm) at 40°C using a linear gradient ramping from 2% B to 40% B in 30 minutes (buffer A: 0.1% formic acid; buffer B: acetonitrile/0.1% formic acid) with a flow rate of 5 μl/min. The column was washed by an increase in 1 min to 80% buffer B that was kept for 6 min. In the next 0.6 min the buffer B composition was changed to 2% and the column was equilibrated for 3 min. For MS calibration of the ion mobility dimension, three ions of Agilent ESI-Low Tuning Mix ions were selected (m/z [Th], 1/ *K*0: 622.0289, 0.9848; 922.0097, 1.1895; 1221.9906, 1.3820). The dia-PASEF windows scheme was ranging in dimension m/z from 400 to 1200 and in dimension 1/*K*0 0:6–1:43, with 32 × 25 Th windows with ramp time 100 ms.

### Quality control samples

To monitor measurement quality and reproducibility, we included 388 WT controls, a strain in which a *his3Δ::kanMX* deletion is complemented by heterologous expression of the *HIS3* enzyme.^15,94^ In addition, we measured 389 quality control (QC) samples (pooled yeast digest, 7 per plate), bringing it to a total of 777 proteome samples measured as controls.

### DIA library generation

The libraries were generated from “gas-phase fractionation”^109^ runs using scanning SWATH^22^ and small precursor isolation windows. 5 μg yeast digests were injected and run on a nanoAcquity UPLC (Waters) coupled to a TripleTOF 6600 (SCIEX) with a DuoSpray Turbo V source (SCIEX). The peptides were separated on a HSS T3 column (Waters, 150 mm × 300 μm, 1.8 μm particles) with a column temperature of 35°C and a flow rate of 5 μl/min. A 55-min linear gradient ramping from 3% acetonitrile/0.1% formic acid to 40% acetonitrile/0.1% formic acid was applied. The ion source gas 1 (nebuliser gas), ion source gas 2 (heater gas), and curtain gas were set to 15 psi, 20 psi, and 25 psi. The source temperature was set to 75°C and the ion spray voltage to 5,500 V. In total 11 injections were run with the following mass ranges: m/z 400–450, 445–500, 495–550, 545–600, 595–650, 645–700, 695–750, 745–800, 795–850, 845–900, 895–1000, and 995–1200. The precursor isolation window was set to m/z 1 except for mass ranges m/z 895–1000 and 995–1200, where the precursor windows were set to m/z 2 and 3, respectively. The cycle time was 3 sec, consisting of high- and low-energy scan, and data were acquired in “high resolution” mode. A spectral library was generated using library-free analysis with DIA-NN directly from these scanning SWATH acquisitions. The UniProt^110^ yeast canonical proteome was used for library annotation.

### Growth assays

Growth assays were performed on SC, SM, and YPD media by time-course imaging of colonies, using our Pyphe pipeline.^111,112^ Library plates were grown from cryostocks in 384 format for three days on agar media. Plates were then multiplexed into 1,536 format on agar with two grids of 96 wild-type controls (complemented *his3Δ* deletion strain) placed in the top-left and bottom-right corners. Plates were then passaged again and copied onto fresh agar plates which were immediately placed into a V800 transmission scanner (Epson) located in an incubator maintained at 30°C. Plates were imaged approximately every 20 min for 40 h. Growth curves based on pixel intensity values were extracted and smoothed using a median and Gaussian filter with kernel sizes of 3. Maximum slopes were then extracted using a sliding window of length 5. Grid values in the bottom-left and top-right corner were extrapolated using linear regression. Maximum slopes were normalised by grid correction^113^ and repeats for the same knock-out were averaged. Assay plates consistently exhibited signal-to-noise ratios above 30 and fractions of unexplained variance below 20%, indicating high data quality.

“Normal” and “slow” growth rates are defined as ≥ 0.8 and < 0.8, respectively (Figure 3A). For the comparison of the dispersion (Figure 3B) we defined the ranges to be more narrow to compare strains with a more defined growth rate and not distributions of growth rates. Here we defined slow growing as normalised growth rates between 0.3 and 0.4 and normal growing as 0.9 to 1.0.

### Quantification and Statistical Analysis

All statistical analyses were done in R.^114^ For basic data manipulation and visualisation the R tidyverse group of packages were used.^95^

Coefficients of variations (CV) were calculated as follows: empirical standard deviations for each protein or precursor were divided by its empirical mean, and are reported in percentages. CV values were calculated for proteins or precursors identified in at least two replicate measurements.

For several analyses, the protein intensities were centred (as mentioned in the respective section). Centred protein intensities were calculated by dividing each protein intensities by the median of the respective protein across all knock-out and WT samples.

Conversion between UniProt IDs, gene names, and open reading frames (ORFs) was done with the bitr function within the clusterProfiler package^115,116^ or using the UniProt database.^110^

For boxplots, the first and third quartiles, as well as the median (thick line), are shown; whiskers extend to the most extreme data point that is no more than 1.5× the interquartile range from the box.

### Normalization, batch correction, filtering, and protein quantification

Raw data processing was carried out with DIA-NN^28^ (Version 1.7.12) with default settings, with MS2 and MS1 mass accuracies set to 20 ppm and scan window size set to 6.

Precursors were filtered for q-values < 0.01 (precursor and protein level) and only proteotypic peptides were considered. Batches (plates) were corrected by bringing median precursor quantities of each batch to the same value (dividing the quantities by the plate median and multiplying them with the median of all plate medians). Precursors were only considered if identified in > 80% of WT samples and if quantified with CV < 50%. Samples were removed if the number of identified precursors was less than 80% of the maximum number of precursors. Protein quantities were obtained using the MaxLFQ algorithm^117^ as implemented in the DIA-NN R package (https://github.com/vdemichev/diann-rpackage). Missing values were imputed with a mixed imputation strategy: Protein quantities that were missing in < 5% of the samples per plate were imputed with a random value between 0 and the minimum protein quantity per plate. Values that were missing in > 5% of the samples per plate were imputed with nearest neighbour averaging (KNN) using the impute.knn function from the R package impute.^98^

### Differential protein expression/abundance analysis

Differential abundance analysis was conducted on the processed data (see above) after log_2_ transformation. We determined differential abundances of proteins in the single-replicate deletion strains by taking into account the variation of each protein in the 388 ild-type replicate measurements across the 57 batches. We used limma^107^ to fit a linear model and applied empirical Bayes for information borrowing between genes, which has proven advantageous on datasets with low numbers of replicates.^107^ The linear models were fitted gene-wise using the lmFit function within the limma package.^107^ Each of the knock-outs was compared against the compendium of 388 wild-type samples using the makeContrasts function (limma R package).^107^ The t-statistics were computed using the ebayes function, allowing an intensity trend in the prior variance (trend = TRUE). Adjusted p-values were extracted using the topTable function. BH was used for multiple testing.^62^ If not mentioned otherwise, we call proteins differentially expressed if the adjusted p-value is below 0.01.

For some analysis, fold-changes were estimated by the ratio of the quantity within a strain and the median quantity of the respective protein across all knock-outs and wild-type strains (centred intensities). Of note, the differences between the medians of the WT samples and the medians of the knock-outs are negligible (ratios of median WT / median KO are < 1.01 and > 0.99).

Strains were not measured in replicates. However, for 145 ORFs, more than one strain exists in the library (these strains have different origins). 141 gene deletions are duplicated and 4 triplicated. For the descriptive analysis (Figure 1), each strain was treated independently in the differential expression analysis. For the functional analysis (enrichments) the duplicated strains were averaged in the differential expression analysis to avoid that the same gene is counted more than once in the overrepresentation analysis.

### Power analysis

In order to estimate the statistical power, we created a simulated dataset that contains simulated WT proteomes (“WT_sim”) as well as one simulated single-replicate KO proteome (“KO_sim”). The proteins in KO_sim and WT_sim are normally distributed. Their standard deviation and mean values were estimated from the measured 388 WT strain proteomes. In order to simulate a biological response in “KO_sim” we changed abundances of 185 randomly assigned proteins (10% of all proteins) and introduced defined fold-changes to the normally distributed values.

First, we evaluated the effect of a varying number of WT strains on the power. We added a fold-change of 0.67/1.5 (log_2_ FC of ±0.58) to 10% of randomly selected proteins and changed the number of “WT_sim”. We then applied the same statistical approach as we used to analyse our dataset (see Differential protein expression/abundance analysis section above). The protein changes we could recall with an adjusted p-value cutoff of 0.01 was 0% for 0–6 WT replicates, 34% for 10 WT replicates, and reached 52% in 21 WT replicates (Figure S1G).

We then repeated the procedure for increasing fold-changes. We used 370 “WT_sim” samples, one “KO_sim” sample, adjusted p-value cutoff = 0.01 (BH), and varied the fold-changes (log_2_ FC between 0.1 and 1 (up and down)) for 185 proteins. We found that for 17%, 48%, and 84% of the proteins, changes could be recalled for log_2_ FC of ±0.3, ±0.5, and ±1.0, respectively (Figure S1H).

Finally we estimated the power for different p-value cutoffs (0.01 to 0.1) using 370 “WT_sim” samples, one “KO_sim” sample, and fixed 0.67/1.5 fold-changes for 185 randomly selected proteins. We could recall 55%, 65%, and 69% of the protein changes with adjusted p-value cutoffs of 0.01, 0.05, and 0.1 (Figure S1I).

### Effect of deletions on functional interactions and networks

Functional interactions were downloaded from YeastNet (v3, Kim et al.^34^) and compared to differential protein expression (p-value < 0.01, BH for multiple testing) upon gene deletion of interaction partners. The total number of affected pairs (interaction partner is DE) within each data type (co-expression, high-throughput protein–protein interaction, genetic interactions, literature-curated protein–protein interaction, phylogenetic profiles, genomic neighbour, co-occurrence, tertiary structure of protein) was divided by the total number of differentially abundant proteins across the dataset and multiplied by 100 (% of differential expression explained by known connection between knock-out and protein) (Figure 2D).

Differentially expressed proteins of distance *i* (from gene deletion) were normalised to the total number of interactions of distance *i* within the respective data type (co-expression, high-throughput protein–protein interaction, genetic interactions, literature-curated protein–protein interaction, phylogenetic profiles, genomic neighbour, co-occurrence, tertiary structure of protein). The number of affected pairs within each distance and data type are illustrated as dot sizes in Figure 2E. Significance was calculated with a one-sided hypergeometric test (more significantly affected interactions than random) using the phyper function within the stats R package.^114^ Some interactions are represented in more than one network, but the average overlap between two networks is less than 10% (Figure S2B).

### Analysis of paralogs (ohnologs)

The assignment of paralogs from whole genome duplications (ohnologs) was downloaded from the yeast gene order browser^35^ (see key resources table). The impact of a deletion on an ohnolog partner was estimated by using the differential expression analysis as outlined in the differential expression analysis methods section. We calculated the total number of differentially expressed ohnolog partners (reduced and increased abundance separately) and normalised it to the average number of protein changes (in percent). The statistical significance was calculated with a hypergeometric test (statistical significance of having more protein abundance changes among paralog pairs) (Figure 2F). To calculate the covariation of ohnolog pairs we calculated Spearman correlation coefficients for all assigned pairs. The significance was calculated with a Wilcoxon signed rank test. For the analyses of duplicated metabolic enzymes, we obtained the list and the classification from Kuepfer et al.^36^ The groups “partial backup” and “specialised” were not considered, as less than 3 measured proteins or knock-outs could be assigned to those groups. We further grouped paralogs as protein components of the ribosome (according to the GO term “structural constituent of ribosome”^37^ in Figures 2H and 2I.

### Growth-rate associated proteins

Growth association of proteins was evaluated by calculating the correlation coefficients of growth rates with protein abundance changes across the KO strains. The cor function within the stats R package^114^ was used and Pearson correlation coefficients were reported.

### Analysis of chromosomal copy-number alterations

For each strain, log_2_ ratios between protein abundances and the median expression of the respective protein across all KO strains (presumed euploid) were calculated. Log_2_ expression ratios were then normalised strain-wise by subtracting the median log_2_ ratio per KO strain from all log_2_ protein ratios. To find aneuploid strains, chromosomes were assessed in 100-kb windows, with iteration of the start of these windows in 10-kb steps. If protein abundances for at least five proteins within a window had been measured, the median segment log_2_ ratios were calculated. A strain was considered potentially aneuploid if it contained at least one window with a median log_2_ ratio > 0.5. Manual inspection of chromosome-ordered log_2_ ratios of these suspected aneuploids was performed in order to verify the strains as whole-chromosome or segmental aneuploids and to exclude strains falsely predicted to be aneuploid after the above described filtering. Heatmaps were generated with the ComplexHeatmap R package and default settings.^103^

Enrichment analysis was performed on the knock-outs that induced aneuploidy using the GO slim terms^37^ (Figure S3G). The run-GSAhyper function (Fisher’s exact test) within the piano R package^105^ was used. BH was used for multiple testing.^62^ All measured knock-outs were used as background.

### Machine-learning models for the prediction of protein half-lives and ribosome occupancy

We used elastic net regression models^55^ and tested if the abundance changes of a protein across the knock-outs can predict ribosome occupancy (as a proxy of translation rate) and protein half-life. To construct the elastic net models, protein abundance values measured across the knock-outs were used as predictor variables and the protein half-lives or ribosome occupancies from reference datasets^56,57^ as response variables. The generalised linear models with elastic net^55^ were applied using the glmnet implementation^118,119^ within the caret R package.^97^ We used elastic net models, because its penalty is particularly useful for correlated or high numbers of predictor variables.^118^ The data were log_2_ transformed (protein quantities and half-lives/ribosome occupancy), scaled, and centred. Models were trained using the train function (caret R package^97^). 10-fold cross-validation with a tune length of 5 was performed for parameter optimisation. The models were trained on 80% of the proteins (1,398 proteins for half-life; 1,392 proteins for ribosome occupancy) and subsequently applied on the remaining 20% of the proteins (348 proteins for half-life; 346 proteins for ribosome occupancy). The protein abundances across all measured knock-out strains were used as predictor variables (n = 4,552). Plots and R squared values were reported for proteins from the test set (not used for parameter optimisation). Feature/variable importance was estimated using the absolute value of the coefficients corresponding to the tuned model, as implemented in the varimp function within the caret R package.^97^

Enrichment on the features for the ribosomal profiling data was done using features/variables (knock-outs) with a relative importance > 30. Gene set analysis (Fisher’s exact test) was performed using the runGSAhyper function within the piano R package.^105^ The GO slim terms^37^ were used as geneset. BH was used for multiple testing.^62^ All measured knock-outs were used as background.

We used reference datasets for protein turnover, obtained by metabolic labelling^57^ as well as ribosome occupancy, determined by ribosomal profiling.^56^ For the latter, the mean values of RepA and RepB from the mixed parental ribosome occupancy (reference dataset^57^) was used as an estimate of ribosome occupancy.

### Systematic analysis of complex subunit alterations

A list of protein complexes was downloaded from the EBI complex portal.^87–89^ Complexes with less than 3 measured proteins were excluded from the analysis. In addition, the following complexes were removed before the analysis due to redundancy in sub-units: CPX-1882, CPX-1883, CPX-776, CPX-1675, CPX-473, CPX-1602, CPX-769, CPX-770, CPX-771, CPX-776, CPX-581, CPX-44, CPX-32, CPX-1102. Further, we filtered out knock-outs where we detected the knocked-out protein (Figures S1D–S1F). In total we considered 51 complexes. Statistical testing was performed by comparing the complex subunits between the respective knock-outs and wild-type samples (n = 264), assuming that the subunits have equal variances. Non-parametric testing was performed using a Wilcoxon signed-rank test with an adjusted p-value cutoff of 0.05. BH was used for multiple testing correction.^62^ In Figure 5B, complexes were considered as affected if at least one knock-out of a subunit showed significant differential expression (adj. p-value < 0.05) of the measured proteins.

### Genome-scale pathway perturbation map

The KO strains were grouped according to KEGG pathways.^69,70^ Differential expression analysis was performed using the limma approach (see section differential protein expression/abundance analysis), but instead of the knock-outs (as above), the pathways were defined in the model and compared against the wild type using the makeContrasts function within the limma R package.^107^ The results of this differential expression analysis (p-value < 0.01, BH for multiple testing^62^) were fed into an over-representation analysis.

Gene set analysis (Fisher’s exact test) was performed using the runGSAhyper function within the piano R package.^105^ The adjusted p-value cutoff was set to 0.01 (BH was used for multiple testing^62^). KEGG terms^69,70^ were used as gene sets and the minimum and maximum gene set size were set to 5 and 100, respectively. All measured knock-outs were used as backgrounds. The genome-scale pathway perturbation map was illustrated as a chord diagram using the chordDiagram function within the circlize R package.^104^ Arrows face from perturbed pathways (as grouped for the differential abundance analysis) to the affected pathways (significantly enriched terms).

### Functional enrichment analysis of PP, RPP, PS, PC

Enrichment analysis for groups of KO strains and proteins was performed using Gene Ontology (GO), KEGG,^70^ and Reactome^90^ terms. To test enrichment of proteome profiles of KO strains (PP), we considered the group of differentially abundant proteins in each strain, defined as those with a BH-adjusted p-value < 0.01 from the limma analysis. The same strategy was used to test the groups of KO strains in which a protein was differentially expressed (RPP). The KOs that were strongly linked to a KO-of-interest based on proteome profile similarity were defined as those that scored in the top 1% of all analysed KO–KO associations (PS). The proteins that were strongly linked to a protein-of-interest by protein covariation were defined as those that scored in the top 1% of all analysed protein–protein associations (PC). The TopGO R package was used to test GO term enrichment in these groups using the default “weight01” algorithm, which takes the GO topology into account.^106,120^ The nodeSize parameter was set to 10, which prunes the GO hierarchy from the terms which have less than 10 annotated genes. TopGO terms with a p-value of 0.01 or lower were considered to be enriched. GO annotations for yeast were obtained from the website of the Gene Ontology consortium (see key resources table). KEGG and Reactome – based gene set enrichment analysis (Fisher’s exact test) was performed using the runGSAhyper function within the piano R package.^105^ The minimum and maximum gene set size were set to 10 and 100, respectively. The adjusted p-value cutoff was set to 0.01 (BH was used for multiple testing^62^). Only the knock-outs and proteins subjected to the PP, RPP, PS, and PC analyses, respectively, were used as background for the functional enrichment analysis (rather than the entire yeast genome or proteome).

### Enrichments within the TCA cycle

Enrichments were performed as described above for the genes belonging to the KEGG term “citrate cycle (TCA cycle).”^69,70^ We tested only for the enrichments of the same pathway and therefore no multiple testing was applied. P-value cutoff was set to 0.01 (Figure 6G).

### Data transformation for the analysis of protein covariation and proteome profile similarity

For proteome profile similarity assessment of KO strains, protein intensities were divided by the median intensity across all strains (WT, KO, and QC samples) and log_2_ transformed. The resulting data matrix contained relative protein level changes of 1,850 proteins across 5,463 samples without missing values (see above for imputation strategy). For protein covariation analysis, protein intensities were transformed in the same way but starting from a non-imputed and less stringently filtered data matrix (considering precursors identified in > 50% rather than 80% of WT samples), because this type of analysis is not affected by a moderate amount of missing values.^121^ The resulting data matrix contained 2,292 proteins across 5,552 samples (includes WT and QC samples) with 7.5% missing values.

### Profile comparisons using correlation and distance metrics

To avoid spurious correlations between proteome profiles, log_2_ fold-changes were normalised such that the median fold-change of each protein across KOs was zero. To avoid spurious correlations between KO profiles, log_2_ fold-changes were normalised such that the median protein fold-change of each KO was zero. We tested a range of similarity metrics, including three correlation metrics, three “conventional” distance metrics (Euclidean, Manhattan, Minkowski), and two decision-tree-based distance metrics. Input data were scaled (z-transformed) prior to calculation of conventional distance metrics. Pearson and Spearman correlations, as well as Euclidean, Manhattan, and Minkowski distances were calculated using base R functions. Biweight midcorrelation (bicor) was applied through the WGCNA R package.^101,122^ The treeClust R package^96^ was used to calculate distances with the treeClust algorithm, using default parameters except for minsplit = 500, which had been identified as the optimal parameter setting using PR test runs. Unsupervised random forests (uRFs) were used through the randomForest R package.^99^ Note that uRFs do not work on datasets with missing values, so for covariation analysis via uRFs, missing values were imputed using the *k-*nearest-neighbour imputation algorithm of the impute R package.^98^

The topological overlap matrix was calculated using the TOMsimilarity function of the WGCNA R package.^100,101^

### Precision-recall analysis

Precision–recall (PR) curves and the areas under these curves were calculated using the PRROC R package.^102^

We used two separate, partially overlapping gold standards for the PR analyses in this study: one based on functional protein–protein associations reported by String v11^73^ and one based on the COMPLEAT set of protein complexes.^74^ For the STRING gold standard, true positive (TP) associations were defined as gene pairs with a combined STRING score of ≥ 700 (high confidence). False positive (FP) pairs were defined as all pairs that were not linked by STRING at any confidence level. The COMPLEAT gold standard was described previously.^15,74^ From both gold standards we further excluded FP pairs that had been found associated by either String, COMPLEAT, BioGRID v3.5^91^ or Gene Ontology.^123^ In addition, we removed all genes that had not been detected as part of the Y5K dataset, and those that could not be unambiguously cross-mapped between UniProt IDs and systematic gene names (OLNs). The resulting gold standards contain 70,023 unique String TPs, 58,785 unique COMPLEAT TPs, and 14,726 TPs that overlap between the two standards.

### Feature selection for gene-function prediction

As an initial proof-of-principle experiment, we subjected 50 randomly selected groups of 185 proteins to PR analyses using the STRING gold standard. Although this was only a miniscule fraction of the theoretically possible 5×10^259^ 185-protein combinations, several of these randomly selected subsets identified functionally related knock-out genes with higher precision than a PR analysis using all 1,850 proteins. This indicates that the high dimensionality of our data was a challenge (“curse of dimensionality”) and that functional predictions could be improved by selecting an optimal subset of proteins (feature selection). We therefore aimed to systematically select the best features (i.e. proteins) to link KO strains. In principle, it would be possible to identify the optimal subset of features for this task simply by selecting those that result in the largest area under the PR curve. However, such a “cherry-picking” approach may not extrapolate well to other data sets or gold standards. We therefore based our feature selection process on the prediction of growth rates. Our rationale was that proteins which are important for growth-rate prediction may also be the ones whose expression changes are relevant for linking KO strains (see legend of Figure S6 for additional explanations).

For this feature selection process we took advantage of the ability of random forests (RFs) to determine the importance of individual features (i.e. proteins) for a regression task.^124^ We used the randomForest R package^99^ to train RF regression models on the growth rates of all KO and wildtype strains. We trained three separate RFs (technical replicates) for each of the three growth media (SC, SM, YPD) for which growth rates had been measured. These 9 RF models were created using default parameters except for *nodesize*, which was set to 100 to speed up the calculation. To test if RF regression models can accurately predict growth rates, we created a 10th model in which we withheld 500 strains from the training set and predicted their growth rates in YPD medium (Figure S3A).

Feature importance was determined as the increase in node purity for each protein (“IncNodePurity” output from the RF models). Under the chosen parameter settings we found this measure of feature importance to be highly reproducible between technical replicates, i.e. RF models trained on the same input data (R^2^ = 0.99). However, feature importance differed considerably for growth rate predictions in the three growth media (e.g. R^2^ = 0.65 between SM and SC). Feature importances from different RF models were scaled (z-transformed) and proteins were ranked by the minimum importance they achieved across different RF models.

To select the best features (KO strains) for protein covariation analysis, KO strains were ranked by the number of differentially expressed proteins in decreasing order. The most responsive 10% of KO strains selected in this way proved to be the ideal set of KO strains to use for protein covariation analysis (Figure S6).

### UMAP visualization

The R implementation of the Uniform Manifold Approximation and Projection (UMAP) algorithm^77,125,126^ was used to reduce protein and KO correlation matrices down to two dimensions. Since UMAP uses distances and not similarities to calculate the low dimensional projection of the data, biweight midcorrelations were inverted (multiplied by –1) before UMAP analysis.

### Additional data annotation

Essential yeast genes were defined as those annotated as “inviable” in the Saccharomyces Genome Database.^92^ A list of uncharacterised yeast genes was downloaded from YeastMine.^127^ Protein lengths were extracted from UniProt.^110^ Protein abundances for Figure S5F, which had to cover proteins that were not detected in this analysis, were extracted from a meta-analysis of absolute protein concentrations in yeast.^93^ Gene Ontology (GO) term enrichment for the *vma5Δ* and *rtc2Δ* strains (Figure 7) were carried out using the Panther website as described.^128^

### Comparison with genetic interactions

For the in-depth comparison of our data with genetic interactions (GIs) we considered genome-scale genetic interaction scores and genetic interaction profiles from Costanzo and colleagues.^78^ Raw scores from the Nonessential x Nonessential (NxN), Essential x Essential (ExE) and Nonessential x Essential (ExN) networks were downloaded from https://thecellmap.org/costanzo2016/ and the duplicate pairs were averaged. For GI profiles we considered the similarity values (Pearson correlations) computed by Costanzo et al. for all gene pairs combined, available from the same website. For the purpose of our precision–recall analysis, all gene pairs with a genetic interaction score (ε) > 0 were considered to be positive GIs, and those with ε < 0 were defined as negative GIs. Interactions involving an essential gene, i.e. those from the ExE or ExN networks, were further distinguished from interactions between non-essential genes from the NxN network. Precision–recall analysis was performed as described above.

## Supplementary Material

Table S1-S5

Table S6

Table S7

Supplemental figures

## Data Availability

Raw mass spectrometry data have been deposited to the ProteomeXchange Consortium (http://proteomecentral.proteomexchange.org) via the massIVE repository with the dataset identifier ProteomeXchange: PXD036062. The dataset identifier is listed in the key resources table. The measured growth rates and the processed datasets derived from the raw data have been deposited at Mendeley Data and the link is listed in the key resources table. The data are additionally available through an interactive web application: https://y5k.bio.ed.ac.uk/. This paper contains analyses that used existing, publicly available data. The identifiers for the datasets are also listed in the key resources table. No custom software codes were generated as part of this study. All analyses conducted in R, using standard, publicly accessible packages obtained either through GitHub (https://github.com/), the Comprehensive R Archive Network (CRAN, https://cran.r-project.org/), or Bioconductor (https://www.bioconductor.org/). Any additional information required to reanalyze the data reported in this paper is available from the lead contact upon request.

## Highlights

- Proteomes were recorded for 4,699 non-essential gene deletions in *S. cerevisiae*
- Proteomic responses reflect general protein properties and functional relationships
- Protein abundance changes depend on turnover, complexes, growth, and genome structure
- Functional proteomics reveals gene function in four complementary strategies

## In brief

By combining functional genomics with proteomics, molecular phenotypes in the yeast *Saccharomyces cerevisiae* can be assigned at genome scale, and systems-level insights reveal principles of how gene function relates to protein expression.
