# Supplementary material for: The proteomic landscape of genome-wide genetic perturbations
Source: Cell. Author manuscript; Available in PMC 2024 Feb 16. (PMC7615649; doi:10.1016/j.cell.2023.03.026)
Supplement: Table S1-S5 [file EMS193941-supplement-Table_S1_S5.pdf]

Cell, Volume *186*

Supplemental information

The proteomic landscape of genome-wide genetic perturbations

Christoph B. Messner, Vadim Demichev, Julia Muenzner, Simran K. Aulakh, Natalie Barthel, Annika Röhl, Lucía Herrera-Domínguez, Anna-Sophia Egger, Stephan Kamrad, Jing Hou, Guihong Tan, Oliver Lemke, Enrica Calvani, Lukasz Szyrwiel, Michael Mülleder, Kathryn S. Lilley, Charles Boone, Georg Kustatscher, and Markus Ralser

Table S1, related to STAR methods. Timing and mobile-phase composition for the applied non-linear chromatography gradient.

Time (min)	Mobile phase (% A)
initial	97
0.86	92.9
2.42	88.8
5.53	84.7
9.38	80.6
13.02	76.4
15.48	72.3
17.27	68.2
19	60
20	20
20.5	20
21.5	97
27.5	97

Table S2, related to STAR Methods. Lower and upper m/z limits of precursor isolation windows in the applied variable window SWATH-MS method.

Lower m/z	Upper m/z
399.5	410.4
409.4	420.3
419.3	429.7
428.7	439.6
438.6	448.9
447.9	458.8
457.8	468.7
467.7	478.1
477.1	488.5
487.5	499
498	509.4
508.4	519.9
518.9	530.9
529.9	541.9
540.9	552.8
551.8	564.4
563.4	575.9
574.9	588
587	600.1
599.1	612.2
611.2	625.4
624.4	639.2
638.2	652.9

651.9	667.2
666.2	681.5
680.5	696.9
695.9	712.3
711.3	728.8
727.8	747.5
746.5	766.2
765.2	786
785	806.9
805.9	828.9
827.9	854.8
853.8	882.8
881.8	915.3
914.3	957.6
956.6	1015.4
1014.4	1098.4
1097.4	1249.7

Table S3, related to Figure 2. Proteins that are up-regulated upon deletion of their paralogue.

Knock-out strain	Up-regulated protein	Fold change	Adjusted p-value
rps14aΔ	Rps14b	4.085177	6.7E-34
rpl4aΔ	Rpl4b	2.72258	4.6E-04
rpl8aΔ	Rpl8b	1.310114	6.1E-04
rps29bΔ	Rps29a	1.372421	2.3E-05
rps1aΔ	Rps1b	1.534089	1.2E-14
rpl6bΔ	Rpl6a	1.36172	2.4E-04
rnr4Δ	Rnr2	6.540719	5.7E-119
rpl7aΔ	Rpl7b	4.987068	2.3E-48
ura7Δ	Ura8	1.691639	1.6E-09
rps9bΔ	Rps9a	5.445963	4.4E-03
cit1Δ	Cit2	2.337526	5.5E-03
rps7aΔ	Rps7b	1.858272	6.7E-13
rpl16bΔ	Rpl16a	2.150493	5.7E-46
rpl14aΔ	Rpl14b	1.772908	2.6E-06
rpl8bΔ	Rpl8a	2.291356	2.7E-13
tif4631Δ	Tif4632	1.952875	4.1E-03
rps29aΔ	Rps29b	1.473908	7.4E-06
rpl6aΔ	Rpl6b	2.249223	8.2E-39
sse1Δ	Sse2	2.356487	8.0E-06

Table S4, related to Figure 4. The top 15 strains selected by the elastic net model are listed with corresponding feature importance.

Gene name	Feature importance
elp4Δ	100
spt10Δ	89.19691
mot2Δ	88.79057
ubp3Δ	86.14195
def1Δ	84.50996
yjl175wΔ	80.81533
fyv4Δ	80.60749
npr3Δ	78.62733
ykl169cΔ	74.90061
ino1Δ	71.6569
get1Δ	69.56296
blm10Δ	66.9569
bar1Δ	64.10587
seh1Δ	59.31983
ecm30Δ	58.42478

Table S5, related to Figure 4. The top 15 strains selected by the elastic net model are listed below with corresponding feature importance.

Strain	Feature importance
fun30Δ	100
sds24Δ	96.3988
ino1Δ	95.01005
ncs2Δ	88.48427
ybr196c-aΔ	84.96767
dur12Δ	82.29651
gim4Δ	72.66398
mot2Δ	70.20363
ybr220cΔ	69.28264
vps51Δ	59.81237
get1Δ	59.42979
rps11bΔ	56.14677
bud25Δ	55.60507
ydr048cΔ	53.71978
sbe2Δ	53.65612
