# Supplementary material for: The proteomic landscape of genome-wide genetic perturbations
Source: Cell. Author manuscript; Available in PMC 2024 Feb 16. (PMC7615649; doi:10.1016/j.cell.2023.03.026)
Supplement: Supplemental figures [file EMS193941-supplement-Supplemental_figures.pdf]

CellPress
OPEN ACCESS

Supplemental figures

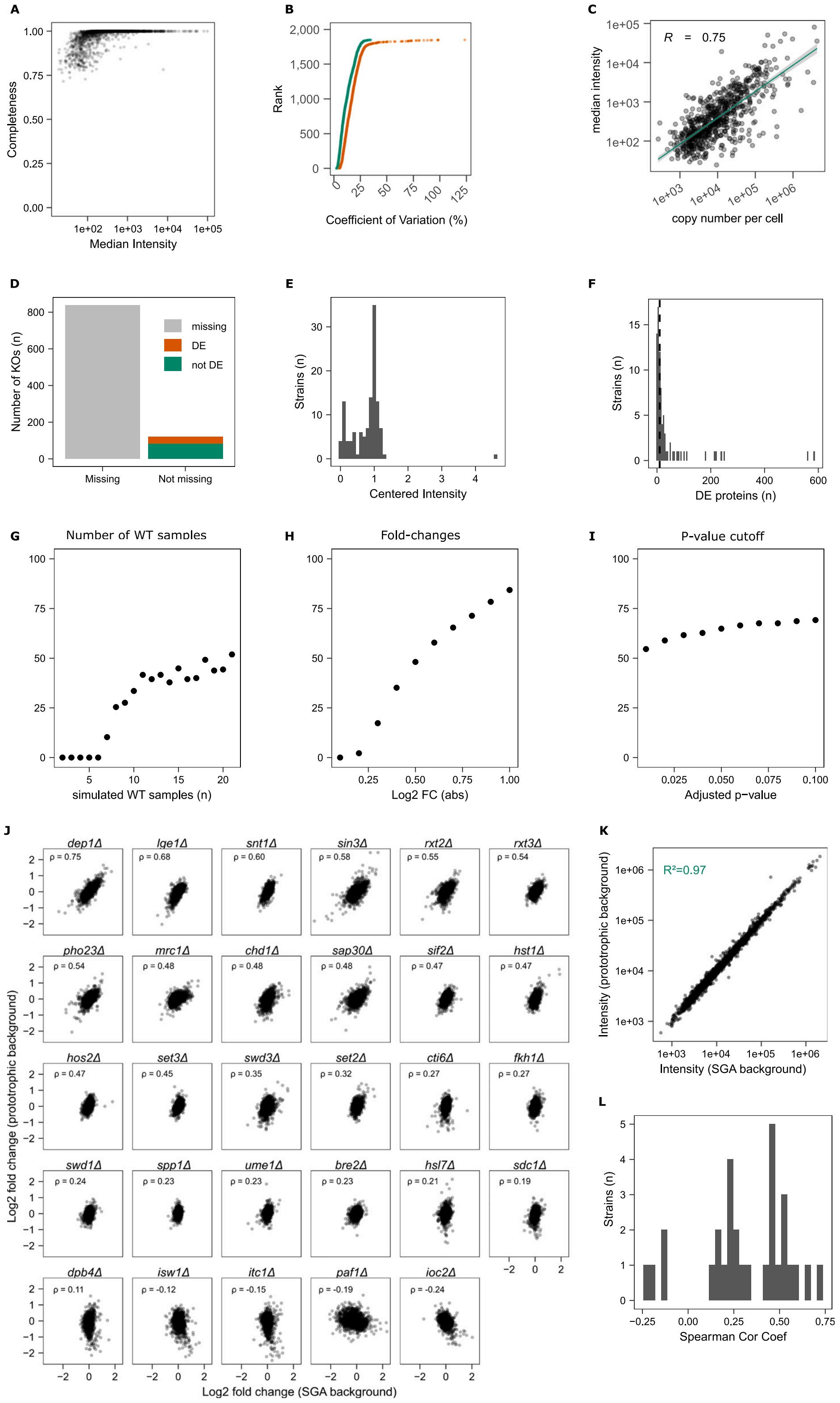

(legend on next page)

Figure S1. Precise quantitative proteomes for the genome-scale yeast gene-deletion collection, grown in a minimal medium, related to Figure 1

(A) Consistency of identifications and its dependency on protein abundance. Completeness was calculated for each protein as the number of samples in which the respective protein was identified divided by the total number of samples. Completeness is plotted as a function of abundance (approximated by the median intensity across KOs). The filtered and processed dataset (no imputation) was used.

(B) The coefficients of variation (in %) were calculated for whole-process control samples (WT, green, n = 388), and KO samples (orange, n = 4,699).

(C) Median intensity values across all WT samples are plotted against copy numbers per cell taken from a reference dataset.[33] Scales are $\log_{10}$ transformed.

(D) In 87% (839) of the testable KO strains, the deleted protein was not detected. In 39 strains (4%) the deleted proteins were found significantly changed in abundance, and in 82 strains (9%) the supposedly deleted protein is detected at a level similar to wild type (not significantly differentially expressed; 0.01 p value cutoff).

(E) Measured intensities of proteins that are deleted but detected (n = 121 strains). In 39 of those strains the protein was found significantly differentially expressed. Intensities are centered (normalized by the median intensity across all KOs). 0.01 p value cutoff, BH for multiple testing.[62]

(F) Number of proteins that are differentially expressed (p value 0.01, BH for multiple testing[62]) in strains with detectable and non-differentially expressed deleted proteins (n = 82 strains). 44 strains have >10 proteins differentially expressed.

(G) Effect of varying number of simulated WT samples on statistical power. We generated a simulated dataset with normally distributed samples, with standard deviation and mean values calculated from the 388 WT samples measured. To simulate a biological response in the “KO_sim” sample we added to 185 proteins (10% of measured proteins; randomly assigned) of this sample (KO_sim) a defined fold-change of 0.67 or 1.5 ($\log_2$ FC of $\pm$0.58). The number of simulated WT samples was varied and we calculated the percentage of proteins we could recall as differentially expressed using a 0.01 adjusted p value cutoff (BH for multiple testing[62]).

(H) Effect of varying fold-changes on statistical power. Same as (G) but with varying fold-changes ($\log_2$ FC between 0.1 and 1). We used 370 “WT_sim” samples, 1 KO_sim sample, adjusted p value cutoff = 0.01 (BH), and varied the $|\log_2 \mathrm{FC}|$ between 0.1 and 1 (up and down) for 185 proteins.

(I) Effect of varying p value cutoffs on statistical power. Same as above but with varying p value cutoffs (adjusted p values between 0.01 and 0.1). We used 370 WT_sim samples, 1 KO_sim sample, and a fixed 0.67-/1.5-fold-change for 185 randomly selected proteins.

(J) Protein responses (fold-changes) upon gene deletions in two different backgrounds (prototroph and synthetic genetic arrays [SGAs]). Fold-changes were calculated by dividing each protein quantity by the median quantity of the respective protein across all the KOs within a background. Spearman correlation coefficients are given and plots are sorted by decreasing coefficients.

(K) Protein intensities of WT samples are compared between prototroph background and SGA[38] mutants. The mean values of 6 samples measured in each background are compared. x axis and y axis were $\log_{10}$ transformed.

(L) Correlation coefficients of protein responses (fold-changes) upon deletions in two different backgrounds are shown as histogram. The pairwise correlations were calculated for 29 different KOs (*dep1Δ*, *lge1Δ*, *snt1Δ*, *sin3Δ*, *rxt2Δ*, *rxt3Δ*, *pho23Δ*, *mrc1Δ*, *chd1Δ*, *sap30Δ*, *sif2Δ*, *hst1Δ*, *hos2Δ*, *set3Δ*, *swd3Δ*, *set2Δ*, *cti6Δ*, *fkh1Δ*, *swd1Δ*, *spp1Δ*, *ume1Δ*, *bre2Δ*, *hsl7Δ*, *sdc1Δ*, *dpb4Δ*, *isw1Δ*, *itc1Δ*, *paf1Δ*, *ioc2Δ*).

CellPress
OPEN ACCESS

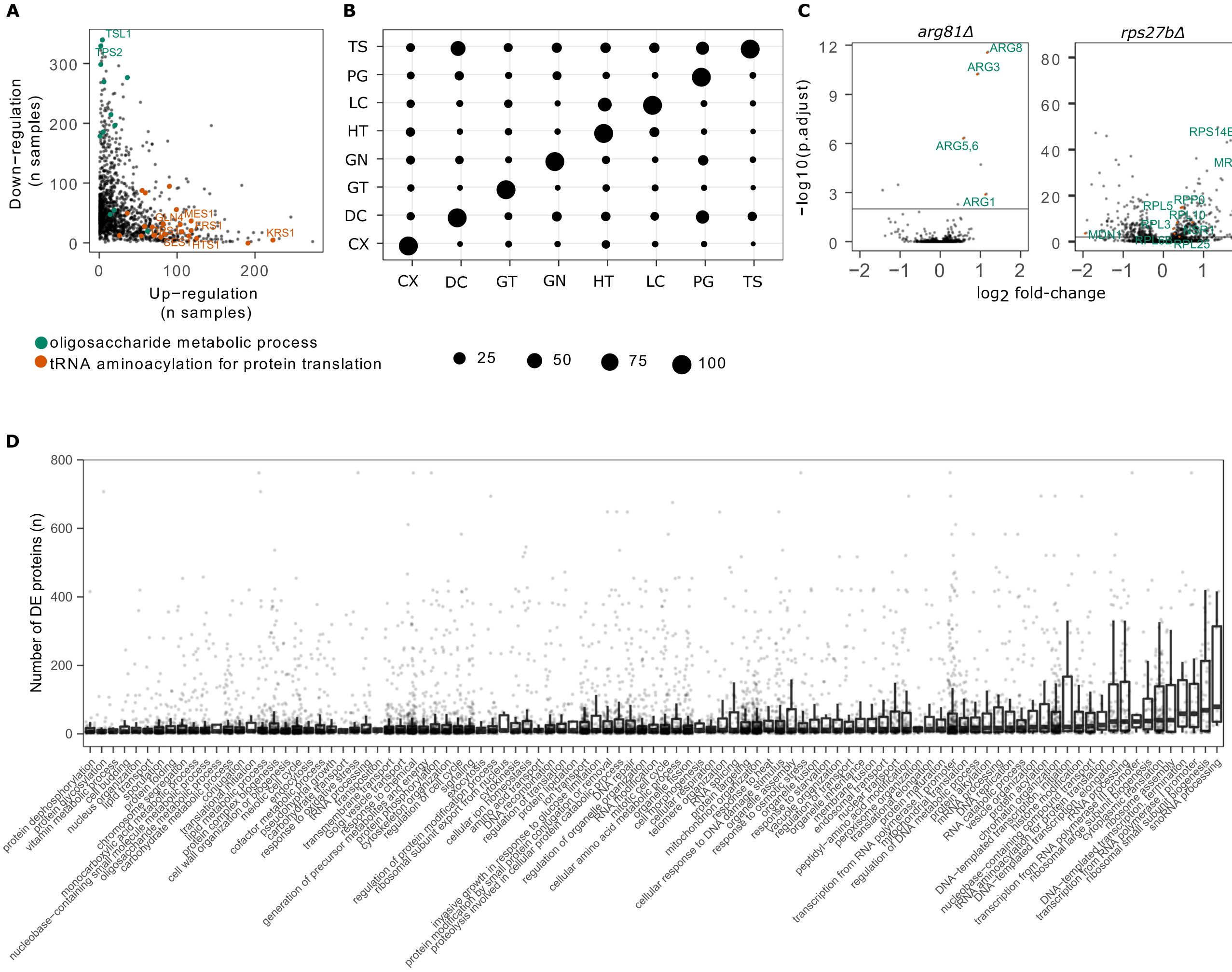

Figure S2. The proteomic response to systematic gene deletion reveals principles of protein abundance changes, related to Figure 2

(A) Upregulations (x axis) and downregulation (y axis) of each protein across the KO strains. Proteins related to oligosaccharide metabolic process (green) and tRNA aminoacylation for protein translation (orange) are labeled.

(B) Overlap between genetic, physical, and functional interaction networks. The overlap is normalized to the size of the network given on the y axis. Numbers are given in %, with 100 indicating a complete overlap. Interactions were downloaded from YestNet (v3, Kim et al.[34]) (LC, literature curated PPI; TS, tertiary structure of protein; HT, high-throughput PPI; GN, genomic neighbor; CX, co-expression; GT, genetic interaction; DC, domain co-occurrence; PG, phylogenetic profiles).

(C) Differential protein expression in *arg81Δ* (left) and *rps27bΔ* (right) strains. While *arg81Δ* has a specific proteome response with a low number of differentially expressed proteins, many proteins are affected in *rps27bΔ*. Differential expression was calculated with the limma package[107] and BH was used for multiple testing[62] (STAR Methods). The x axis shows centered $\log_2$ intensities and the y axis shows adjusted p values ($-\log_{10}$ transformed).

(D) Number of differential expressions for each gene deletion, grouped by Gene Ontology slim terms for "biological process."[37] Differential expression was calculated with the limma package[107] and BH was used for multiple testing[62] (STAR Methods).

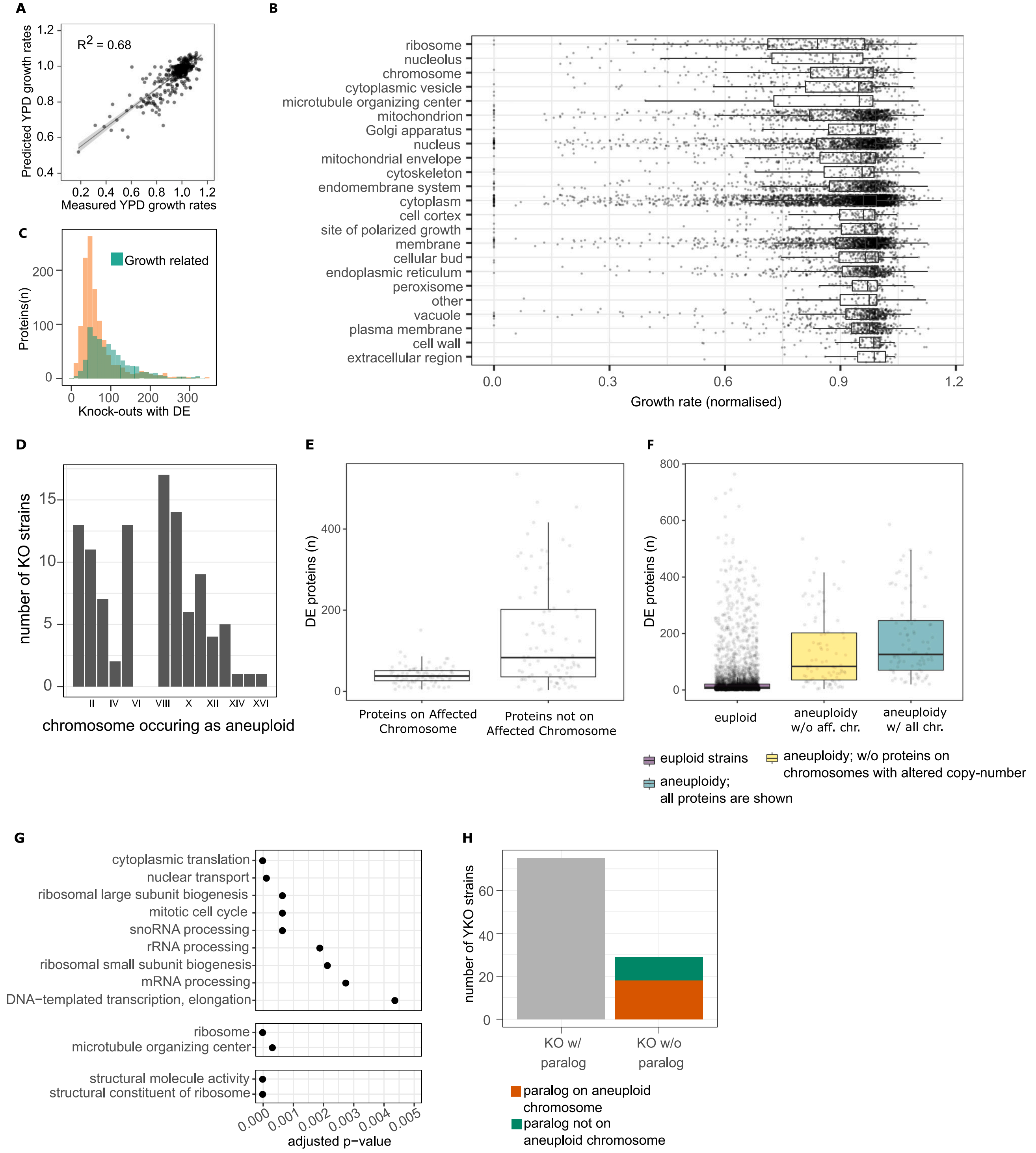

Figure S3. Broad proteomic changes in many slow-growing strains can be explained by chromosomal copy-number variations (aneuploidies) and their transmission to the proteome, related to Figure 3

(A) Growth rates were predicted from the protein abundances using a random forest (RF) algorithm. Growth rates in YPD medium were measured for all strains (STAR Methods). We then trained an RF regression model to predict these KO strain growth rates from the abundances of the 1,850 quantified proteins. 500 KO strains were left out from training the RF regression model, which was subsequently used to predict their growth rates in YPD medium. See also Figure S6 and STAR Methods.

(legend continued on next page)

(B) Growth rates (normalized) for each KO strain, grouped by cellular compartments (GO slim terms for *cellular compartment*[37]). The first and third quartiles, as well as the median, are shown with boxplots, and the whiskers extend to the most extreme data point that is no more than 1.5× the interquartile range from the box.

(C) The proteomic changes in KO strains are only partially explained by growth-rate-correlated proteins. Number of differential expressions (adjusted p value < 0.01, BH for multiple testing[62]) across the KO strain was calculated for each protein, and proteins were grouped into growth-related (green) and non-growth-related proteins (orange) depending on their respective correlation with growth rate (growth-related: $r > 0.2$ or $r < -0.2$, non-growth-related: $-0.2 < r < 0.2$).

(D) Chromosomes differ in their likelihood of being aneuploid in the genome-scale deletion collection.

(E) Number of differentially abundant proteins (adjusted p value < 0.01, BH for multiple testing[62]) is shown for proteins on the chromosome with the copy-number variation (n = 84, median = 38) and for the remaining chromosomes (n = 84, median = 83.5). All strains identified as having whole-chromosome aneuploidy were considered.

(F) The numbers of significantly changed proteins are compared between euploid (n = 4,161, median = 9), aneuploidy without the proteins on the chromosomes with altered copy number (n = 84, median = 83.5), and aneuploidy including the proteins on the chromosomes with altered copy number (n = 84, median = 126). Adjusted p value cutoff < 0.01 (BH for multiple testing correction). The first and third quartiles, as well as the median (thick line), are shown with boxplots; whiskers extend to the most extreme data point that is no more than 1.5× the interquartile range from the box.

(G) Enrichment analysis (hypergeometric test) was performed on the KOs that induced aneuploidy using the GO slim gene sets (BP, MF, and CC).[37] Significant terms (adjusted p value < 0.01) are shown and ranked by significance (decreasing from top to bottom).

(H) Number of aneuploid KOs with and without paralogs. The aneuploid strains with paralogs are grouped into strains where the paralog is on the aneuploid chromosome (orange) and strains where the paralog is not on the aneuploid chromosome (green). Paralogs from whole-genome duplications (ohnologs) were considered and their annotations were downloaded from the Yeast Gene Order Browser[35] (see key resources table).

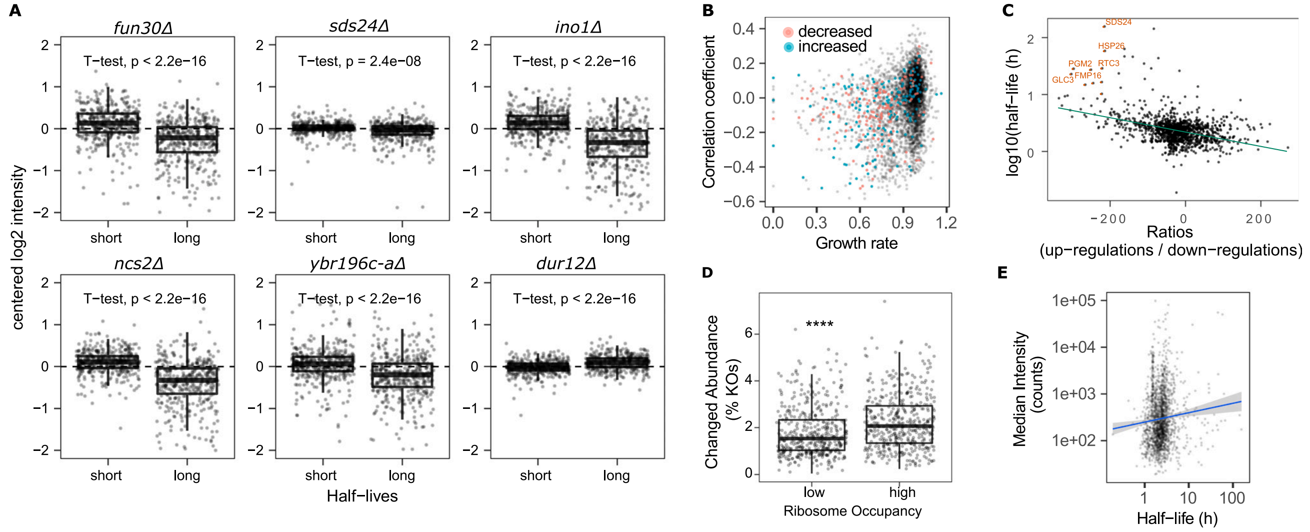

Figure S4. The interdependency of differential protein expression with translation rate and turnover, related to Figure 4

(A) Half-life-dependent protein-abundance changes for the top 6 features (KOs) selected by the elastic net model (*fun30Δ*, *sds24Δ*, *ino1Δ*, *ncs2Δ*, *ybr196c-aΔ*, *dur12Δ*). Protein half-lives[57] (log_2 transformed) are plotted against centered log_2 intensities. Long and short half-lives are defined as being above 3rd quartile and below 1st quartile, respectively.

(B) Unspecific half-life-dependent protein-abundance changes are observed across all growth rates and cell sizes. Correlation coefficients (Pearson) were calculated for all strainwise relationships between protein expression changes and half-lives. Thus, high correlation coefficients indicate a tendency to upregulate long-lived and downregulate short-lived proteins. Correlation coefficients are plotted against the growth rate (normalized), and strains with phenotypes characterized by decreased and increased cell size[37] are colored.

(C) The directionality of differential expression is given as ratios (number of upregulations/number of downregulations) for each protein and is plotted against its protein half-life (y axis). Protein half-lives were log_{10} transformed.

(D) Proteins with higher ribosome occupancies are more often differentially abundant. Low and high ribosome occupancies are defined as proteins with ribosome occupancies shorter or longer as the median of all considered ribosome occupancies. Ribosome occupancies were taken from a reference dataset and were determined by ribosomal profiling.[56] Differential abundance is given as % changed across all measured KOs (differential abundance of a particular protein across the KO/total number of KO × 100).

(E) Protein abundances (intensities) are plotted as a function of half-lives (in h). x axis is log_{10} transformed. Little correlation was observed with $r = 0.09$ (Pearson correlation coefficient) and $p < 0.01$.

The first and third quartiles, as well as the median (thick line), are shown with boxplots; whiskers extend to the most extreme data point that is no more than 1.5× the interquartile range from the box.

CellPress
OPEN ACCESS

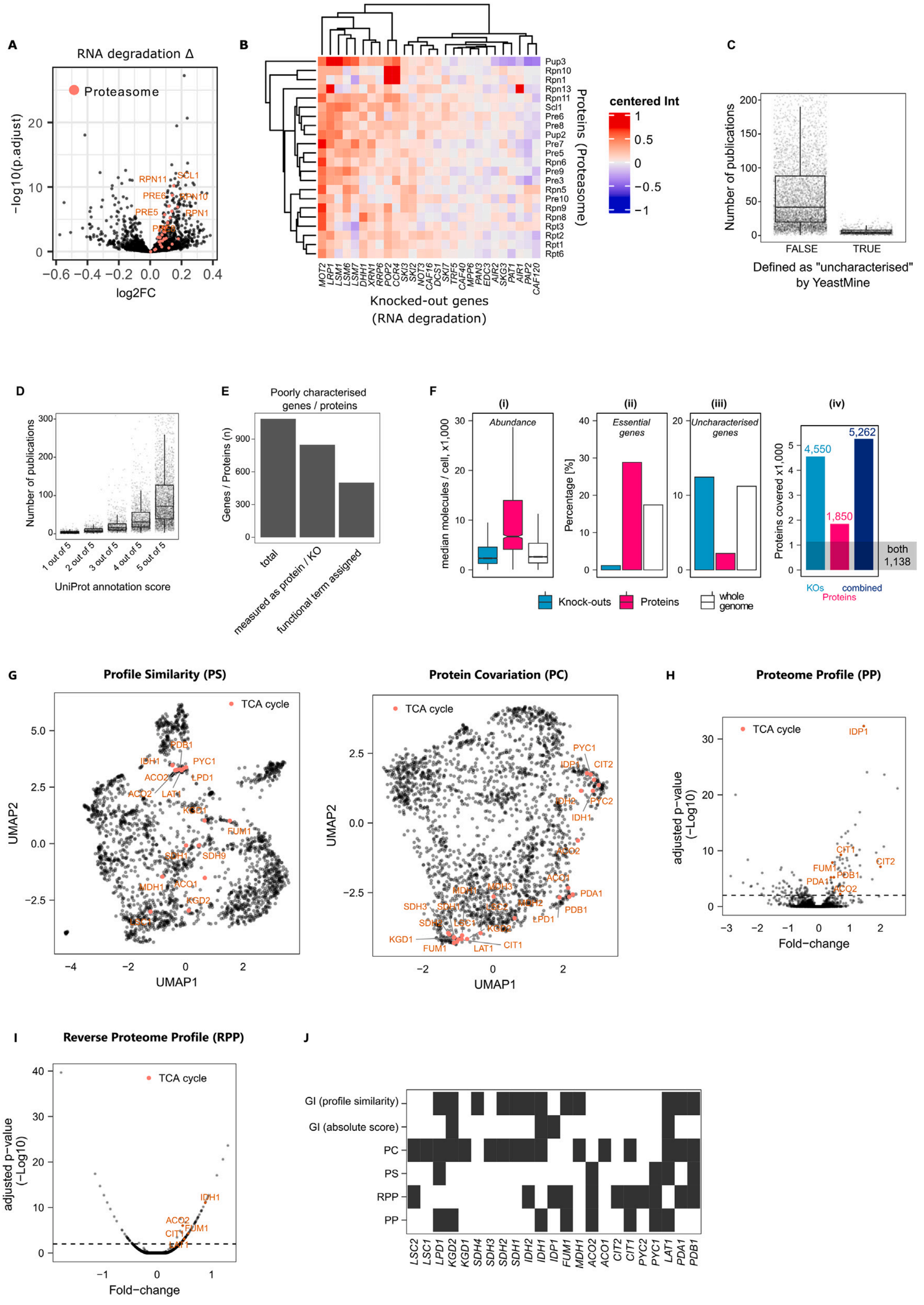

(legend on next page)

Figure S5. Annotating the genome using functional proteomics, related to Figure 6

(A) Differential expression for KOs involved in RNA degradation. KO strains were grouped together according to the KEGG term “RNA degradation”[69,70] (*CAF120*, *PAP2*, *AIR1*, *PAT1*, *SKG3*, *AIR2*, *EDC3*, *PAN3*, *MPP6*, *CAF40*, *TRF5*, *SKI7*, *DCS1*, *CAF16*, *NOT3*, *SKI2*, *SKI3*, *CCR4*, *POP2*, *RRP6*, *XRN1*, *DHH1*, *LSM7*, *LSM6*, *LSM1*, *LRP1*, *MOT2*) and compared to WT samples using the limma package.[107] BH was used for multiple testing.[62] Proteasome proteins are colored. Log_2 fold changes are shown on the x axis; adjusted p values ($-log_{10}$ transformed) on the y axis.

(B) RNA-associated KOs[69,70] (horizontally) and significantly changed proteasomal proteins (vertically). Protein intensities were centered and log_2 transformed.

(C) Many yeast genes are understudied. The number of publications linked to yeast genes according to the *Saccharomyces* Genome Database[37] is shown with boxplots. YeastMine currently classifies 722 proteins as “uncharacterized.” A median of 5 publications can be mapped to these, compared to a median of 42 publications for the remaining genes.

(D) Same data as in (C), but genes were divided based on the annotation score assigned to each gene by UniProt. The 2,913 best-annotated yeast genes (5 out of 5) have a median of 103 publications each, whereas the 468 worst-characterized genes (1 out of 5) have a median of 4 publications.

(E) Poorly characterized genes/proteins captured in our dataset and with our functional annotation strategies. The total number of poorly characterized genes/proteins (UniProt annotation score 1 or 2 or defined as uncharacterized by YeastMine), the number of poorly characterized genes/proteins measured in our dataset and the number of poorly characterized genes/proteins that have at least one functional term assigned by one of the presented strategies (PP, RPP, PS, PC) (STAR Methods).

(F) Gene deletions and high-throughput proteomes capture complementary sets of proteins. Compared to the whole yeast genome, genes covered by mass spectrometry are biased toward more-abundant proteins (Fi) and essential genes (Fii), whereas genes deleted in the KO library are more likely covering low abundant and non-essential proteins that contain also more uncharacterized genes (Fiii). In combination, gene deletions and high-throughput proteomics cover 5,262 unique genes, which is 79% of the yeast genome annotation, and more than can be assessed with either technique in separation. 1,138 genes are covered by both KO and protein quantification (Fiv). Note that the number of essential genes covered by KOs is very small, but not zero. This is because essential genes were defined here as those with an “inviable” phenotype in the *Saccharomyces* Genome Database (STAR Methods), which comprises a few genes that are viable under the conditions used in this study.

(G) UMAPs grouping KO strains by profile similarity (left) and proteins by covariation (right). Genes/proteins that are part of the citrate cycle (TCA cycle) according to the KEGG classification[69,70] are labeled.

(H) Proteome profile of *pyc1Δ* shown as volcano plot. Log_2 fold-changes are shown on the x axes; $-log_{10}$-adjusted p values are shown on the y axes. Differential expression was calculated using the limma R package[107] (STAR Methods).

(I) Reverse proteome profile of *Pyc1* shown as volcano plot. Log_2 fold-changes are shown on the x axes; $-log_{10}$-adjusted p values are shown on the y axes. Differential expression was calculated using the limma R package[107] (STAR Methods).

(J) Functional annotations capture known interactions within the TCA cycle. KEGG enrichment analysis was performed for genes/proteins within the TCA cycle and significant associations with other proteins/genes of the same pathway (TCA cycle) are shown as black squares (p value < 0.01). For PP analysis, the enrichment was performed on the differentially expressed proteins in each strain and for RPP the KOs in which the respective protein was differentially expressed. For PS, PC, genetic interaction scores (absolute values) and profile similarities we considered the highest-scoring 1% of associations in the network. Genetic interactions scores and profiles were taken from Costanzo et al.[78]

CellPress
OPEN ACCESS

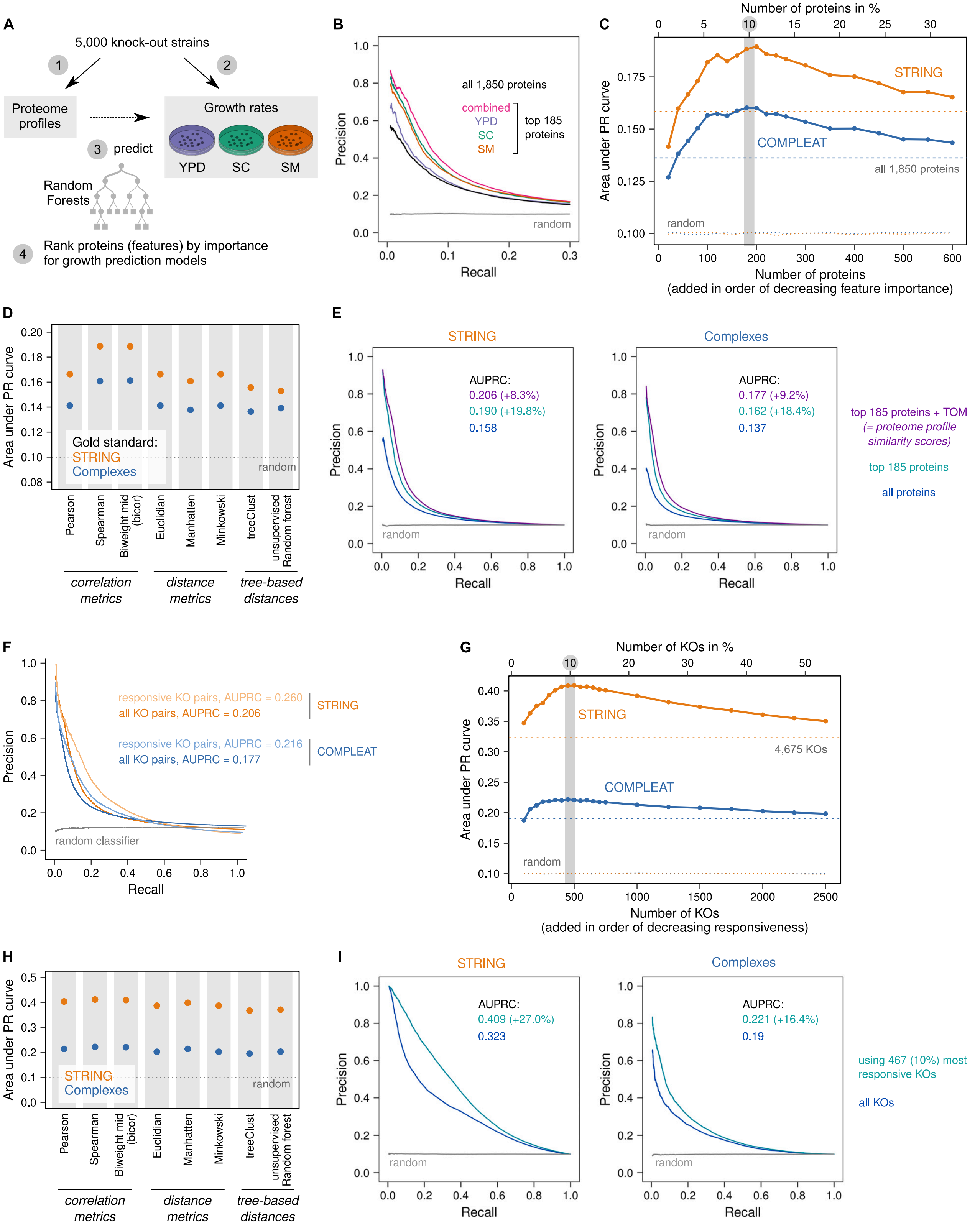

(legend on next page)

Figure S6. Feature selection and optimization of proteome profile similarity and protein covariation assessment, related to STAR Methods

(A) A common strategy for feature selection in data science is the use of random forests (RFs), which offer a straightforward way to assess the importance of each feature for a regression model. We measured the growth rates of the KO strains in three growth media (YPD, SM, SC). We then trained RF regression models to predict these KO strain growth rates from the abundances of the 1,850 quantified proteins. The importance of each feature (protein) for these predictions was extracted from the RF models.

(B) We performed precision-recall (PR) analyses to test if proteins that are important for growth-rate prediction are also useful to identify functionally related KO strains. Indeed, using the 185 proteins with the highest feature importance outperformed the use of all 1,850 proteins. Notably, performance was further improved by combining feature importances across the three growth media, which was achieved by ranking proteins based on the minimal scaled importance they achieved in any RF model. This PR analysis used the STRING gold standard.

(C) To determine the optimal number of features (proteins) to select in this way, proteins were ranked by feature importance (across all three growth media) and a series of PR analyses was performed. The plot shows the areas under the PR curves (AUPRCs), using either STRING or COMPLEAT gold standards. Performance increases as more proteins are added, peaks around 185 proteins (10% of the 1,850 proteins used for this analysis), and then decreases again. This suggests that to compare proteome profile similarities of KO strains it is best to consider only the 10% of proteins with the highest feature importance.

(D) Various correlation and distance metrics were compared by PR analysis for how well they identify profile similarity across the ~5,000 yeast KO strains, on the basis of the 185 pre-selected proteins. Optimal performance is observed for two types of robust correlation metrics, Spearman's correlation and biweight midcorrelation, with the latter becoming our preferred choice as it can be calculated more efficiently.

(E) A topological overlap measure (TOM) further improved the precision with which KO strains of functionally related genes can be linked, as shown by PR analyses using the STRING (left) or COMPLEAT (right) gold standards. Feature selection improves performance by 18.4%–19.2% compared to correlating all 1,850 quantified proteins. Taking into account the topology of the resulting correlation network helps to remove false-positive links and improves performance by an additional 8.3%–9.2%. These TOM-modified biweight midcorrelations of the 185 selected proteins constitute our proteome profile similarity scores.

(F) PR curves showing that focusing the analysis on the 2,290 "responsive" KO strains strongly improves performance. This means the proteome profiles of responsive KOs can be compared more accurately and will therefore lead to better gene-function predictions. A responsive strain is defined here as having more differentially expressed proteins than the median strain.

(G) Feature selection also improved protein covariation analysis. In this case, KOs were ranked by "responsiveness," defined as the number of differentially expressed proteins. PR analyses were performed starting with the 100 most responsive strains and gradually including more strains up until using all 4,675 KO strains that had been included in the limma analysis. Based on the performance peak observed in this way, we proceeded using the 10% (n = 467) most responsive strains to measure protein covariation.

(H) Comparison of metrics capturing protein covariation across the 467 pre-selected KO strains. Spearman's correlation and biweight midcorrelation marginally outperformed other metrics, with the latter again becoming our preferred choice.

(I) PR analyses using STRING and COMPLEAT gold standards, respectively, showing that feature selection improves the detection of functionally related proteins by 16.4%–27% (compared to correlating all ~5,000 yeast strains). Note that in contrast to the proteome-profile-similarity network of KOs, taking into account the topology of the protein covariation network did not improve performance further and was therefore omitted. Consequently, the biweight midcorrelations across the 476 selected KO strains constitute our protein covariation scores.

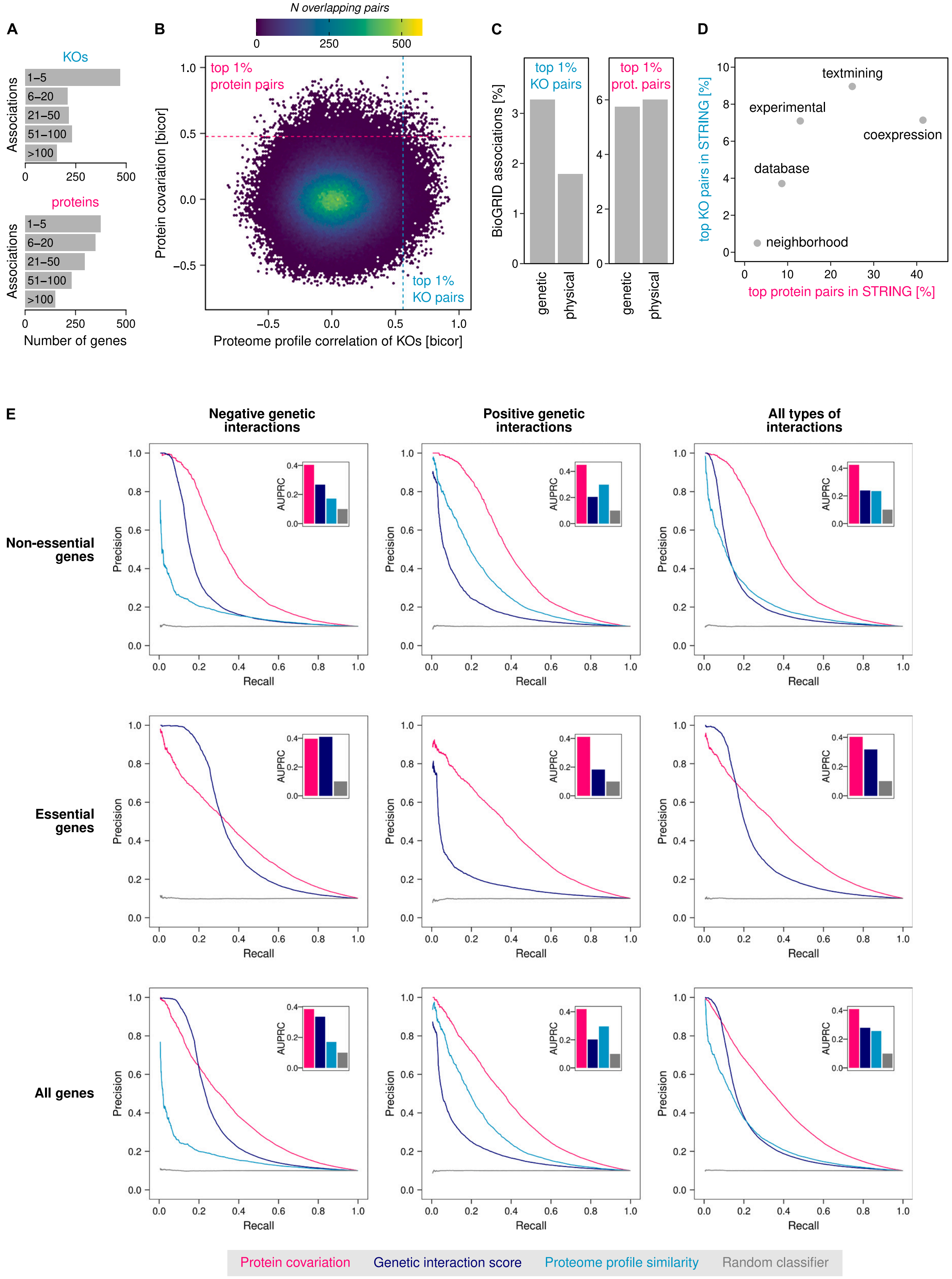

(legend on next page)

Figure S7. Proteome profile similarity and protein covariation are complementary to each other and to previously known functional associations, related to STAR Methods

(A) Breakdown of the highest-scoring 1% of associations across both approaches. These cover 1,284 KOs and 1,396 proteins, respectively.

(B) Biweight midcorrelation (bicor) coefficients of gene pairs that were covered by proteome profile similarity of KO strains and by protein covariation are plotted against each other. There is no common trend and the top 1% associated pairs by each approach overlap only marginally. This shows that the two approaches capture a different set of functional associations among the same set of genes.

(C) Top 1% of associations were mapped to known interactions in BioGRID, showing that pairs detected by KO profile similarity are more likely to have been previously detected as genetic rather than physical interaction. Covarying proteins, on the other hand, are covered better by previously known physical interactions.

(D) The same associations mapped to known functional associations in STRING and broken down by category. Covarying proteins are most similar to (mRNA) co-expression evidence in STRING, whereas proteome profile similarity of KOs best reflects associations found by text mining and experimental assays.

(E) Associations were divided into those involving non-essential and essential genes (rows) and those producing positive and negative genetic interactions (columns). Precision-recall (PR) curves were calculated using the STRING gold standard and the areas under the PR curves (AUPRCs) are shown in the barplot insets. These plots show that proteome profile similarities perform better for positive than negative genetic interactions, and are therefore highly complementary to genetic interaction scores. Protein covariation shows no clear bias for essential vs non-essential genes or for positive vs negative genetic interactions (AUPRC always ~0.4). Genetic interactions scores and profiles were taken from Costanzo et al.[78]
